# *Operando* Converting BiOCl into Bi_2_O_2_(CO_3_)_*x*_Cl_*y*_ for Efficient Electrocatalytic Reduction of Carbon Dioxide to Formate

**DOI:** 10.1007/s40820-022-00862-0

**Published:** 2022-05-03

**Authors:** Huai Qin Fu, Junxian Liu, Nicholas M. Bedford, Yun Wang, Joshua Wright, Peng Fei Liu, Chun Fang Wen, Liang Wang, Huajie Yin, Dongchen Qi, Porun Liu, Hua Gui Yang, Huijun Zhao

**Affiliations:** 1 Centre for Catalysis and Clean Energy, Gold Coast Campus, Griffith University, Gold Coast, QLD 4222 Australia; 2 School of Chemical Engineering, University of New South Wales, Sydney, NSW 2052 Australia; 3 Department of Physics, Illinois Institute of Technology, Chicago, IL 60616 USA; 4 Key Laboratory for Ultrafine Materials of Ministry of Education, School of Materials Science and Engineering, East China University of Science and Technology, Shanghai, 200237 People’s Republic of China; 5 Centre for Materials Science, School of Chemistry and Physics, Queensland University of Technology, Brisbane, QLD 4001 Australia

**Keywords:** Carbon dioxide reduction, Chloride-containing bismuth subcarbonate, Cathodic potential-promoted anion-exchange, Stability

## Abstract

Bismuth-based materials (e.g., metallic, oxides and subcarbonate) are emerged as promising electrocatalysts for converting CO_2_ to formate. However, Bi^o^-based electrocatalysts possess high overpotentials, while bismuth oxides and subcarbonate encounter stability issues. This work is designated to exemplify that the *operando* synthesis can be an effective means to enhance the stability of electrocatalysts under *operando* CO_2_RR conditions. A synthetic approach is developed to electrochemically convert BiOCl into Cl-containing subcarbonate (Bi_2_O_2_(CO_3_)_*x*_Cl_*y*_) under *operando* CO_2_RR conditions. The systematic *operando* spectroscopic studies depict that BiOCl is converted to Bi_2_O_2_(CO_3_)_*x*_Cl_*y*_ via a cathodic potential-promoted anion-exchange process. The *operando* synthesized Bi_2_O_2_(CO_3_)_*x*_Cl_*y*_ can tolerate − 1.0 V versus RHE, while for the wet-chemistry synthesized pure Bi_2_O_2_CO_3_, the formation of metallic Bi^o^ occurs at − 0.6 V versus RHE. At − 0.8 V versus RHE, Bi_2_O_2_(CO_3_)_*x*_Cl_*y*_ can readily attain a FE_HCOO_- of 97.9%, much higher than that of the pure Bi_2_O_2_CO_3_ (81.3%). DFT calculations indicate that differing from the pure Bi_2_O_2_CO_3_-catalyzed CO_2_RR, where formate is formed via a ^*^OCHO intermediate step that requires a high energy input energy of 2.69 eV to proceed, the formation of HCOO^−^ over Bi_2_O_2_(CO_3_)_*x*_Cl_*y*_ has proceeded via a ^*^COOH intermediate step that only requires low energy input of 2.56 eV.

**Supplementary Information:**

The online version contains supplementary material available at 10.1007/s40820-022-00862-0.

## Highlights

- An *operando* synthetic approach was exemplified to enhance catalyst stability for efficient reduction of CO_2_ to formate.
- A highly stable Bi_2_O_2_(CO_3_)_*x*_Cl_*y*_ electrocatalyst was synthesized by direct electrochemical conversion of BiOCl via a cathodic potential-promoted anion-exchange process under *operando* CO_2_RR conditions.
- The surface Cl^−^ in Bi_2_O_2_(CO_3_)_*x*_Cl_*y*_ changes the *p*-orbital electron states to enhance the stability and alters the CO_2_RR pathway to markedly reduce the energy barrier.

**Supplementary Information:**

The online version contains supplementary material available at 10.1007/s40820-022-00862-0.

## Graphical abstract

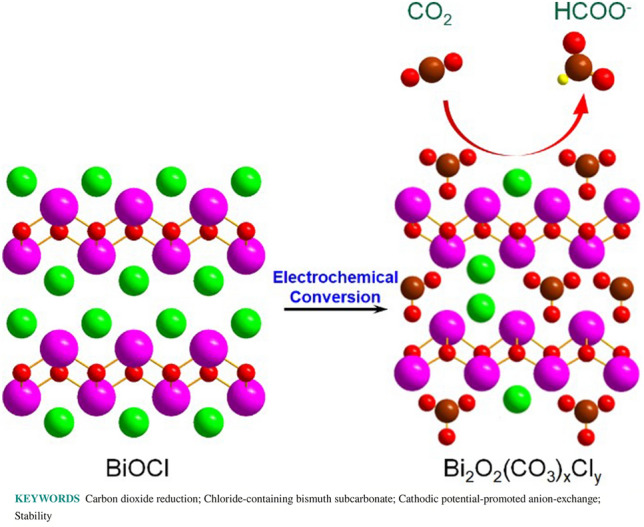

## Introduction

The renewable electricity-powered electrocatalytic carbon dioxide reduction reaction (CO_2_RR) to produce chemicals/fuels not only curbs greenhouse gas emissions but also reduces our reliance on the rapidly diminished petroleum resources [1]. In this regard, various C_1_ (e.g*.*, carbon monoxide, formate, methane and methanol), C_2_ and C_2+_ (e.g*.*, ethylene, ethanol, acetylene, acetate, acetaldehyde, oxalic acid and *n*-propanol) CO_2_RR products have been obtained [2, 3]. Among them, CO and HCOO^−^/HCOOH are the most energy-efficient CO_2_RR products as they can be formed by transferring two electrons to CO_2_. Comparing to CO, converting CO_2_ to HCOO^−^/HCOOH is more desirable because HCOO^−^/HCOOH are more valuable commodity chemicals [4, 5]. To date, the reported high-performance electrocatalysts for CO_2_ reduction to HCOO^−^/HCOOH are almost exclusively made of *p*-block metals-based materials such as In, Pb, Sn, Sb and Bi [6, 7].

Owning to their low toxicity and high selectivity toward HCOO^−^/HCOOH, Bi-based CO_2_RR electrocatalysts have attracted increasing attentions [8, 9]. Various Bi-based CO_2_RR electrocatalysts such as metallic Bi^o^, oxides and subcarbonate (Table S1) have been employed to electrocatalytically convert CO_2_ to HCOO^−^/HCOOH. As shown in Table S1, in general, the metallic Bi^o^-based ones perform better than other forms of bismuth-containing electrocatalysts. Nevertheless, the metallic Bi^o^-based electrocatalysts usually require high overpotentials, consequently the high cathodic potentials, to achieve their optimal performances [10, 11], undesirable for energy efficiency. In addition, high cathodic potentials are favorable for the competing hydrogen evolution reaction (HER), which often leads to low Faradic efficiencies toward HCOO^−^/HCOOH (FE_HCOO_-/FE_HCOOH_) [12]. The bismuth oxides-based electrocatalysts were also reported (Table S1). Noticeably, such electrocatalysts often encounter stability issues because the bismuth oxides in these electrocatalysts can be easily converted to metallic Bi^o^ under CO_2_RR conditions [13]. For example, Deng et al. reported a Bi_2_O_3_ electrocatalyst with the optimal performance at − 0.9 V (vs RHE) to achieve a FE_HCOO_- of 91% with a partial HCOO^−^ current density (*J*_HCOO_-) of ~ 8 mA cm^−2^ [14]. However, the as-synthesized Bi_2_O_3_ is found to be partially converted to metallic Bi^o^ under the CO_2_RR conditions at − 0.9 V vs RHE. In fact, the reported bismuth oxides electrocatalysts require cathodic potentials ≥ − 0.9 (vs RHE) to concurrently achieve FE_HCOO_- > 90% with *J*_HCOO_- ≥ 15 mA cm^−2^ [13, 15, 16]. Under such CO_2_RR conditions, the bismuth oxides in these electrocatalysts are either partially or completely converted to metallic Bi^o^. Other than metallic Bi^o^ and bismuth oxides, Zhang’s group reported the use of ultrathin bismuth subcarbonate (Bi_2_O_2_CO_3_) nanosheets to catalyze CO_2_ reduction to HCOO^−^ [17]. Their Bi_2_O_2_CO_3_ electrocatalyst exhibits a very low overpotential of 610 mV and can achieve a FE_HCOO_- of 85% with a *J*_HCOO_- of ~ 11 mA cm^−2^ at − 0.7 V (vs HRE), however, partial conversion of Bi_2_O_2_CO_3_ to the metallic Bi^o^ occurs within 30 min under − 0.65 V (vs RHE).

As reviewed above, under the required cathodic potentials to concurrently achieve high FE_HCOO_- and *J*_HCOO_-, the reported bismuth oxide and subcarbonate electrocatalysts are unavoidably reduced to metallic Bi^o^, leading to the structural and compositional changes under *operando* CO_2_RR conditions. Critically, such *operando* structural transformation processes are progressive and potential-dependent, leading to great difficulties to confirm the actual active sites, hence the catalysis mechanisms. Parenthetically, the synthetic conditions of the reported bismuth oxide and subcarbonate electrocatalysts are vastly different to their electrocatalytic application conditions, which could be a cause of their structural transformation under the *operando* CO_2_RR conditions. If this is true, the severe *operando* stability issues might be effectively mitigated by employing identical synthesis and application conditions.

In this contribution, we report an approach to electrochemically convert bismuth oxychloride (BiOCl) into chloride-containing bismuth subcarbonate (Bi_2_O_2_(CO_3_)_*x*_Cl_*y*_) under *operando* CO_2_RR conditions (at − 0.8 V vs RHE in CO_2_-saturated 0.5 M KHCO_3_ solution) and use it to exemplify that the *operando* synthesis can be an effective means to enhance the *operando* electrochemical stability of electrocatalysts. Systematic *operando* spectroscopic studies were conducted to depict the conversion mechanism and electrochemical stability. BiOCl is converted to Bi_2_O_2_(CO_3_)_*x*_Cl_*y*_ via the cathodic potential-promoted anion-exchange process. The obtained Bi_2_O_2_(CO_3_)_*x*_Cl_*y*_ can tolerate − 1.0 V versus RHE, while for the wet-chemistry synthesized pure tetragonal phased Bi_2_O_2_CO_3_, the formation of metallic Bi^o^ occurs at − 0.6 V versus RHE, signifying a markedly improved electrochemical stability. No notable structural change and performance decay are observed when Bi_2_O_2_(CO_3_)_*x*_Cl_*y*_ is subjected to the stability test at − 0.8 V versus RHE over a 20 h period. At − 0.8 V versus RHE, Bi_2_O_2_(CO_3_)_*x*_Cl_*y*_ can readily attain a FE_HCOO_- of 97.9%, much higher than that of Bi_2_O_2_CO_3_ (81.3%). The density functional theory (DFT) calculations indicate that differing from Bi_2_O_2_CO_3_-catalyzed CO_2_RR, where HCOOH is formed via a ^*^OCHO intermediate step that requires a high energy input energy of 2.69 eV to proceed, the formation of HCOOH over Bi_2_O_2_(CO_3_)_*x*_Cl_*y*_ has proceeded via a ^*^COOH intermediate step that requires a notably reduced energy input of 2.56 eV.

## Experimental and Calculation

### Materials and Chemicals

Bismuth nitrate pentahydrate (Bi(NO_3_)_3_·5H_2_O, 99%), potassium chloride (KCl, 99.5%), ethanol (C_2_H_5_OH, 99%) and ethylene glycol (C_2_H_6_O_2_, 99.8%) were purchased from Chem-Supply. Urea (CH_4_N_2_O), Nafion (5 wt%) was purchased from Sigma-Aldrich. Carbon paper (TGP-H-060) and Nafion 115 proton exchange membrane were purchased from Alfa Aesar. The carbon paper was ultrasonically treated in deionized water and ethanol, followed by emerging in the concentrated HNO_3_ at 100 °C for 12 h, thoroughly washed with the deionized water and ethanol and dried in air.

### Synthesis of BiOCl-NSs

0.164 g of KCl and 0.868 g of Bi(NO_3_)_3_·5H_2_O were dissolved in 70 mL H_2_O and stirred for 1 h. The solution was transferred into a 100 mL Teflon-lined stainless-steel autoclave and kept at 120 °C for 24 h. The obtained BiOCl-NSs was adequately washed with deionized water and ethanol and dried at 60 °C for 6 h in vacuum oven.

### Synthesis of Bi_2_O_2_(CO_3_)_***x***_Cl_***y***_

Twenty milligrams of the as-synthesized BiOCl-NSs was mixed with 80 μL Nafion solution (5 wt%) and dispersed 0.92 mL isopropanol under sonication for 40 min to form the ink. 100 μL ink was then cast onto the pre-treated carbon fiber paper substrate with an exposed area of 1 × 1 cm^2^ (2 mg cm^−2^ of BiOCl-NSs). The carbon fiber paper with loaded BiOCl-NSs was used as the working electrode and subjected to − 0.8 V (vs RHE) in CO_2_-saturated 0.5 M KHCO_3_ solution for 2 h to electrochemically transform BiOCl-NSs to Bi_2_O_2_(CO_3_)_*x*_Cl_*y*_.

### Synthesis of Bi_2_O_2_CO_3_

For comparative purpose, pure Bi_2_O_2_CO_3_ was synthesized. Under constant stirring, 0.234 g of Bi(NO_3_)_3_·5H_2_O was dissolved into 10 mL H_2_O, followed by adding 1.502 g of CH_4_N_2_O and 10 mL of C_2_H_5_OH. The resultant solution was then placed in the oil bath under 90 °C for 4 h. The obtained pure Bi_2_O_2_CO_3_ was adequately washed with deionized water and ethanol and dried in a vacuum oven of 60 °C for 6 h.

### Electrochemical Measurements

The electrochemical measurements were performed using a Nafion 115 proton exchange membrane separated two-compartment electrochemical cell consisting of a three-electrode system controlled by an electrochemical station (CHI 660E). For CO_2_RR, the Bi_2_O_2_(CO_3_)_*x*_Cl_*y*_ working electrode (1 × 1 cm^2^) was fabricated by operando electrochemical transformation of the immobilized BiOCl-NSs on carbon fiber paper, while the Bi_2_O_2_CO_3_ working electrode was prepared by immobilizing 2 mg cm^−2^ of Bi_2_O_2_CO_3_ on carbon fiber paper (1 × 1 cm^2^). For all electrochemical measurements, an Ag/AgCl (3.5 M KCl) reference electrode, a Pt mesh counter electrode and CO_2_-saturated 0.5 M KHCO_3_ electrolyte (pH of 7.2) were employed. During CO_2_RR, the electrolyte in the cathode compartment was constantly stirred at a rate of 800 rpm and bubbled with CO_2_ at a flow rate of 5 mL min^−1^ controlled by a universal flow meter (Alicat Scientific, LK2). All reported potentials were converted to the reversible hydrogen electrode (RHE) in accordance with *E*_RHE_ = *E*_Ag/AgCl_ + 0.059 × pH + 0.205. The gas chromatography (GC, RAMIN, GC2060) equipped with a flame ionization detector (FID) and a thermal conductivity detector (TCD) was used to qualitatively and quantitatively determine the gaseous products (e.g., H_2_ and CO or other gaseous hydrocarbons). The CO and H_2_ Faradaic efficiency were calculated as below:

1 $${\text{FE}}_{{{\text{CO}}}} = \, 2x_{{{\text{CO}}}} p{\text{GF}}/{\text{IRT}}$$

2 $${\text{FE}}_{{{\text{H2}}}} = \, 2x_{{{\text{H2}}}} p{\text{GF}}/{\text{IRT}}$$

where *x*_CO_ and *x*_H2_ (vol%) are the volume fractions of CO and H_2_ in the exhaust gas, *I* (A) is the steady-state current, *G* = 5 mL min^−1^ is the CO_2_ flow rate, *p* = 1.013 × 10^5^ Pa, *T* = 273.15 K, *F* = 96,485 C mol^−1^, *R* = 8.3145 J mol^−1^ K^−1^.

^1^H nuclear magnetic resonance (^1^H‐NMR) was used to qualitatively and quantitatively determine the liquid phase products, including HCOOH. After reaction, 0.5 mL electrolyte from the cathode compartment was mixed with 0.1 mL D_2_O containing 3-(trimethylsilyl)propanoic acid (TMSP) as the internal standard and subjected to NMR analysis. The Faradaic efficiency for formation of HCOO^−^ was calculated as below:

3 $${\text{FE}} = \, 2F \times n_{{{\text{HCOO}}}} - /\left( {I \times t} \right).$$

### Characterizations

The morphologies and structures of the samples were characterized by SEM (JEOL JSM-7100) and TEM (Tecnai F20, 200 kV). The STEM images were recorded using a probe corrected JEOL JEM-ARM200F instrument with at an acceleration voltage of 200 kV. AFM measurements were performed using a Bruker Dimension Icon system. XRD patterns were collected from a Bruker D8 diffractometer. The *operando* XRD patterns were recorded using a Bruker D8 diffractometer and a home-made three‐electrode electrochemical cell. Raman spectra were taken by a RENISHAW mVia Raman Microscope using a 532 nm excitation laser. The *operando* Raman studies were performed on a RENISHAW mVia Raman Microscope equipped with a microscopic lens immersed under the electrolyte to capture Raman signals and a home-made three‐electrode electrochemical cell consisting of a BiOCl-NSs or Bi_2_O_2_CO_3_ working electrode, an Ag/AgCl (3.5 M KCl) reference electrode and a Pt mesh counter electrode. The working electrodes were prepared by immobilizing BiOCl-NSs or Bi_2_O_2_CO_3_ on a commercial Si substrate (1 × 1 cm^2^). XPS spectra were recorded by Kratos Axis ULTRA using the C1* s* at 284.8 eV as the internal standard. C and O K-edge XAS measurements were performed at the Soft X-ray spectroscopy beamline at Australian Synchrotron Facility, Australia's Nuclear Science and Technology Organisation (Clayton, Victoria, Australia). Bi L_3_-edge XAS measurements were performed at the 10-ID-B beamline of the Advanced Photon Source (APS), Argonne National Laboratory (ANL). Data reduction, processing and subsequent modeling were performed using the Demeter XAS software package [18]. Modeling of the EXAFS data of Bi_2_O_2_(CO_3_)_*x*_Cl_*y*_ was performed using Bi–O, Bi–C and Bi–Bi backscattering paths from the crystal structure of Bi_2_O_2_CO_3_ [19], while the Bi–Cl contributions were generated from the optimized structure generated from the DFT calculations. All EXAFS fitting was performed using an S_0_^2^ value of 0.868, which were obtained by modeling the EXAFS of a reference Bi foil (L_3_-edge at 13,419 eV). To minimize error in CN and NND values, Debye–Waller factors were optimized in initial rounds of EXAFS fitting and then held constant.

### DFT Calculations

All computation studies were performed using density functional theory (DFT) implemented in the Vienna Ab-initio Simulation Package (VASP) code in this study [20, 21]. For the effects of electron–electron exchange and correlation, the Perdew–Burke–Ernzerhof (PBE) functional at the generalized gradient approximation (GGA) level was employed [22]. The projected augmented wave (PAW) potentials were used throughout for ion–electron interactions [23], with the 5*d*^10^6*s*^2^6*p*^3^, 2*s*^2^2*p*^2^, 2*s*^2^2p^4^, 3*s*^2^3*p*^5^ and 1*s*^1^ treated as valence electrons of Bi, C, O, Cl and H, respectively. The plane-wave cutoff of 520 eV was set for all the computations. The (1 × 2) clean {001} faceted Bi_2_O_2_CO_3_ was modeled by a 14-atomic layer slab separated by a vacuum layer of 20 Å in this study. When geometries of all structures were optimized, top seven layers of the surfaces including adsorbate were relaxed, while the bottom seven layers were fixed. The gamma-centered Monkhorst–Pack *k*-point meshes with a reciprocal space resolution of 2*π* × 0.04 Å^−1^ were utilized for structural optimization. For the calculations on CO_2_ and formic acid molecules, a (20 × 20 × 20) Å^3^ unit cell and a Γ-only *k*-point grid were used. All atoms were allowed to relax until the Hellmann–Feynman forces were smaller than 0.01 eV Å^−1^, and the convergence criterion for the electronic self-consistent loop was set to 10^–5^ eV. The adsorption energy of each adsorbate [Δ*E* (eV/*n*)] was calculated as follows:

4 $$\Delta E = \frac{1}{n} \left( {E_{{\text{ad/surf}}} - E_{{{\text{surf}}}} - nE_{{{\text{ad}}}} } \right)$$

where *E*_ad_, *E*_surf_ and *E*_ad/surf_ are the energies of an adsorbate, the clean {001} facet and the surface with adsorbates, respectively. And n is the number of adsorbates on the surface.

Based on computational hydrogen electrode (CHE) model [24, 25], each electrochemical reaction step can be regarded as a simultaneous transfer of the proton–electron pair as a function of the applied potential. The reaction mechanism of CO_2_ reduction should consist of the following elementary reactions:

5 $${\text{CO}}_{2} \left( {\text{g}} \right) + * + {\text{H}}^{ + } \left( {{\text{aq}}} \right) + {\text{e}}^{ - } \to{^{*}\text{COOH}}$$

6 $${^{*}{\text{COOH}}} + {\text{H}}^{ + } \left( {{\text{aq}}} \right) + {\text{e}}^{ - } \to {\text{HCOOH}} + *$$

or

7 $${\text{CO}}_{2} \left( {\text{g}} \right) + * + {\text{H}}^{ + } \left( {{\text{aq}}} \right) + {\text{e}}^{ - } \to{^{*}{\text{OCHO}}}$$

8 $${^{*}{\text{OCHO}}} + {\text{H}}^{ + } \left( {{\text{aq}}} \right) + {\text{e}}^{ - } \to {\text{HCOOH}} + *$$

where * means the corresponding surface and adsorbed states. The free energy for all intermediate states and non-adsorbed gas-phase molecule is calculated as:

9 $$G = E -_{{{\text{elec}}}} + E_{{{\text{ZPE}}}} + \int {C_{p} {\text{d}}T - TS}$$

where the *E*_elec_ is the electronic energy obtained from DFT calculation; *E*_ZPE_ is the zero-point vibrational energy estimated by harmonic approximation; *∫C*_p_d*T* is the enthalpic correction and *TS* is the entropy. Here, reported values of *E*_ZPE_, *∫C*_p_d*T* and *TS* are adopted [24]. The solvation effect has been considered for *COOH by stabilizing 0.25 eV [24].

## Results and Discussion

### Synthesis and Characterization of Bi_2_O_2_(CO_3_)_***x***_Cl_***y***_

**Fig. 1:**
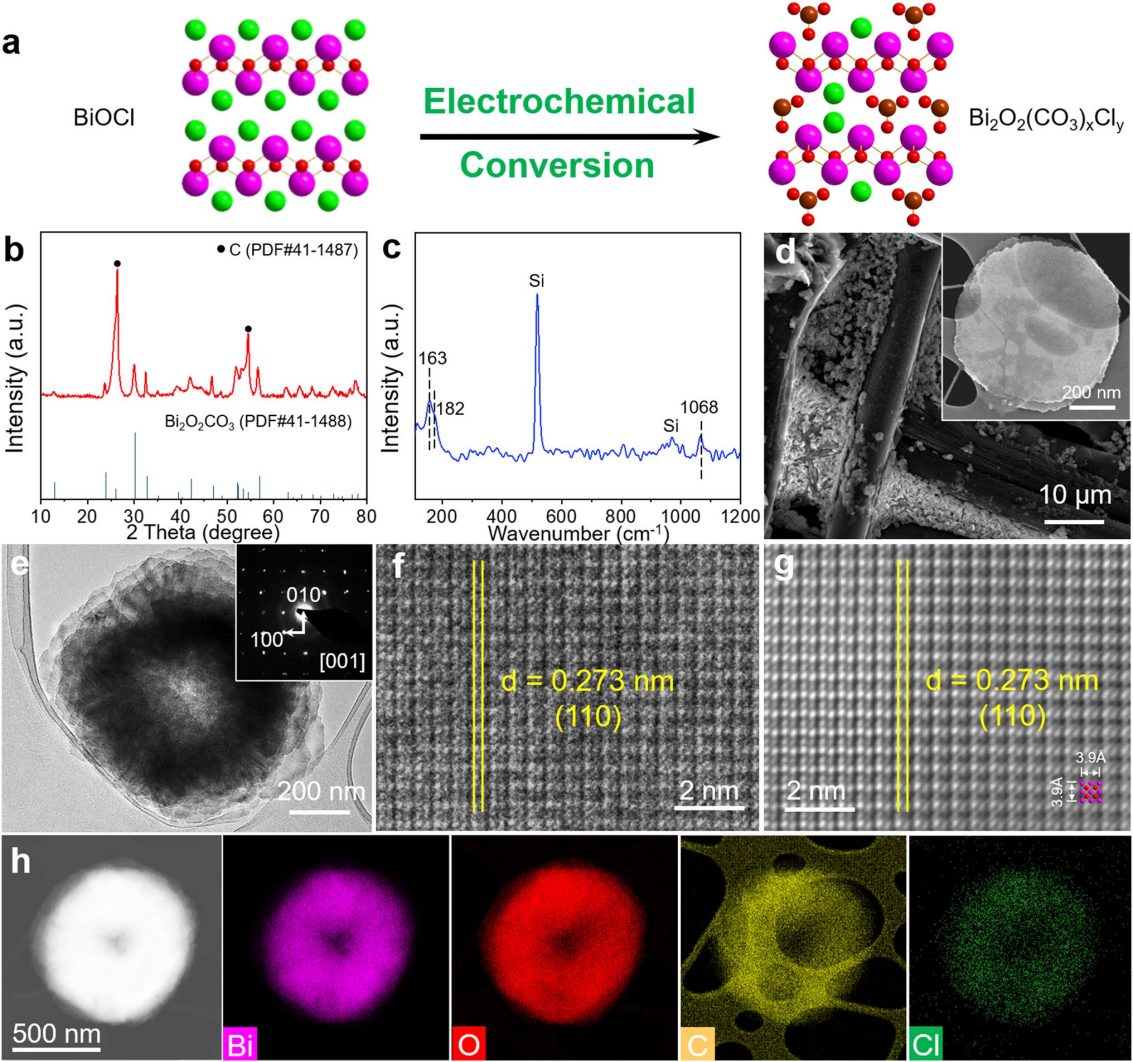
**a** Schematic illustrating electrochemical conversion of BiOCl to Bi_2_O_2_(CO_3_)_*x*_Cl_*y*_ (Bi: pink, O: red, C: brown, Cl: green). **b** XRD pattern, **c** Raman spectrum, **d** FE-SEM images, **e** TEM image and SAED pattern, **f** HRTEM image, **g** IFFT-HRTEM image and **h** HAADF-STEM image and corresponding EDX element mapping images of Bi_2_O_2_(CO_3_)_*x*_Cl_*y*_ resulted from the electrochemical treatment of BiOCl-NSs under − 0.8 V versus RHE in CO_2_-saturated 0.5 M KHCO_3_ solution for 2 h. (Color figure online)

In this work, Bi_2_O_2_(CO_3_)_*x*_Cl_*y*_ was synthesized by direct electrochemical conversion of the pre-synthesized BiOCl under *operando* CO_2_RR conditions (Fig. 1a). The BiOCl nanosheets (BiOCl-NSs) were firstly synthesized as the precursor via a one-pot hydrothermal method [26]. The X-ray diffraction (XRD) pattern of the as-synthesized BiOCl-NSs (Fig. S1) can be indexed to the tetragonal BiOCl (PDF No. 06-0249). The Raman spectrum (Fig. S2) displays two strong peaks centered at 143 and 199 cm^−1^, assignable to $A_{1g}^{1}$ (external) and $A_{1g}^{2}$ (internal) Bi–Cl vibration modes, respectively, while the weak peak at 400 cm^−1^ can be attributed to $B_{g}^{1}$ mode [27]. The atomic force microscopy (AFM) and field-emission scanning electron microscopy (FE-SEM) images (Fig. S3a, b) disclose that the obtained BiOCl-NSs are octagonal shaped with sizes between 600 and 800 nm and thicknesses of ~ 150 nm. The high-resolution inverse fast Fourier transformation transmission electron microscopy (IFFT-TEM) image perpendicular to the nanosheet plane (Fig. S3c) reveals lattice spacings of 2.75 and 2.75 Å with an interplanar angle of 90°, corresponding to the (110) and (1͞10) facets of BiOCl. The selected area electron diffraction (SAED) pattern (Fig. S3d) coincides to the diffraction pattern of the single crystal BiOCl (*k*±*l*0, *k* = *l* = *n*) from [001] zone axis. The aberration-corrected high-angle annular dark-field scanning transmission electron microscopy (HAADF-STEM) image and the corresponding energy-dispersive X-ray spectroscopy (EDX) elemental mapping images confirm the homogeneous distribution of Bi, O and Cl throughout the entire BiOCl-NSs (Fig. S3e).

The as-synthesized BiOCl-NSs were then immobilized onto a conductive carbon paper substrate (1.0 × 1.0 cm^2^) with a loading density of 2.0 mg cm^−2^ (Fig. S4) and subject to − 0.8 V (vs RHE) for 2 h in CO_2_-saturated 0.5 M KHCO_3_ solution to electrochemically convert the loaded BiOCl-NSs into Bi_2_O_2_(CO_3_)_*x*_Cl_*y*_. The XRD pattern (Fig. 1b) of the resultant Bi_2_O_2_(CO_3_)_*x*_Cl_*y*_ can be assigned to the tetragonal phased Bi_2_O_2_CO_3_ (PDF No. 41–1488). The Raman spectrum (Fig. 1c) displays two strong peaks at 163 and 1068 cm^−1^, attributing to the external vibration of Bi_2_O_2_CO_3_ crystal and the *ν*_1_ mode of the intercalated CO_3_^2−^ between the (BiO)_2_^2+^ planes [28, 29]. Raman peak at 182 cm^−1^ could be assigned to the *A*_1g_ mode of the intercalated Cl^−^ in the interlayer [29, 30]. The FE-SEM and AFM images (Figs. 1d and S5) unveil that Bi_2_O_2_(CO_3_)_*x*_Cl_*y*_ possesses a sheeted structure with lateral sizes of 600–800 nm and thicknesses of 130–140 nm. The TEM image (Fig. 1e) shows that Bi_2_O_2_(CO_3_)_*x*_Cl_*y*_ is formed by multiple thin-layer structures with “doughnuts-like” shape, resulting from the substitution of chloride by carbonate. The SAED pattern normal to the nanosheets (inset of Fig. 1e) manifests the reflections of Bi_2_O_2_CO_3_ (*k*00 and 0*l*0, *k* = *l* = *n*) with [001] zone axis. The high-resolution TEM image (HRTEM, Fig. 1f) displays a lattice spacing of 0.273 nm, corresponding to Bi_2_O_2_CO_3_ (110) plane, which is also confirmed by the high-resolution IFFT-HRTEM image (Fig. 1g). The HAADF-STEM image and the corresponding EDX elemental mapping (Fig. 1h) unveil the homogenously distributed Bi, O, C and Cl. The EDX estimated Bi/Cl atomic ratio in Bi_2_O_2_(CO_3_)_*x*_Cl_*y*_ is 17.7:1 (Fig. S6), significantly higher than that of BiOCl (1.2:1), confirming the presence of Cl in Bi_2_O_2_(CO_3_)_*x*_Cl_*y*_.

**Fig. 2:**
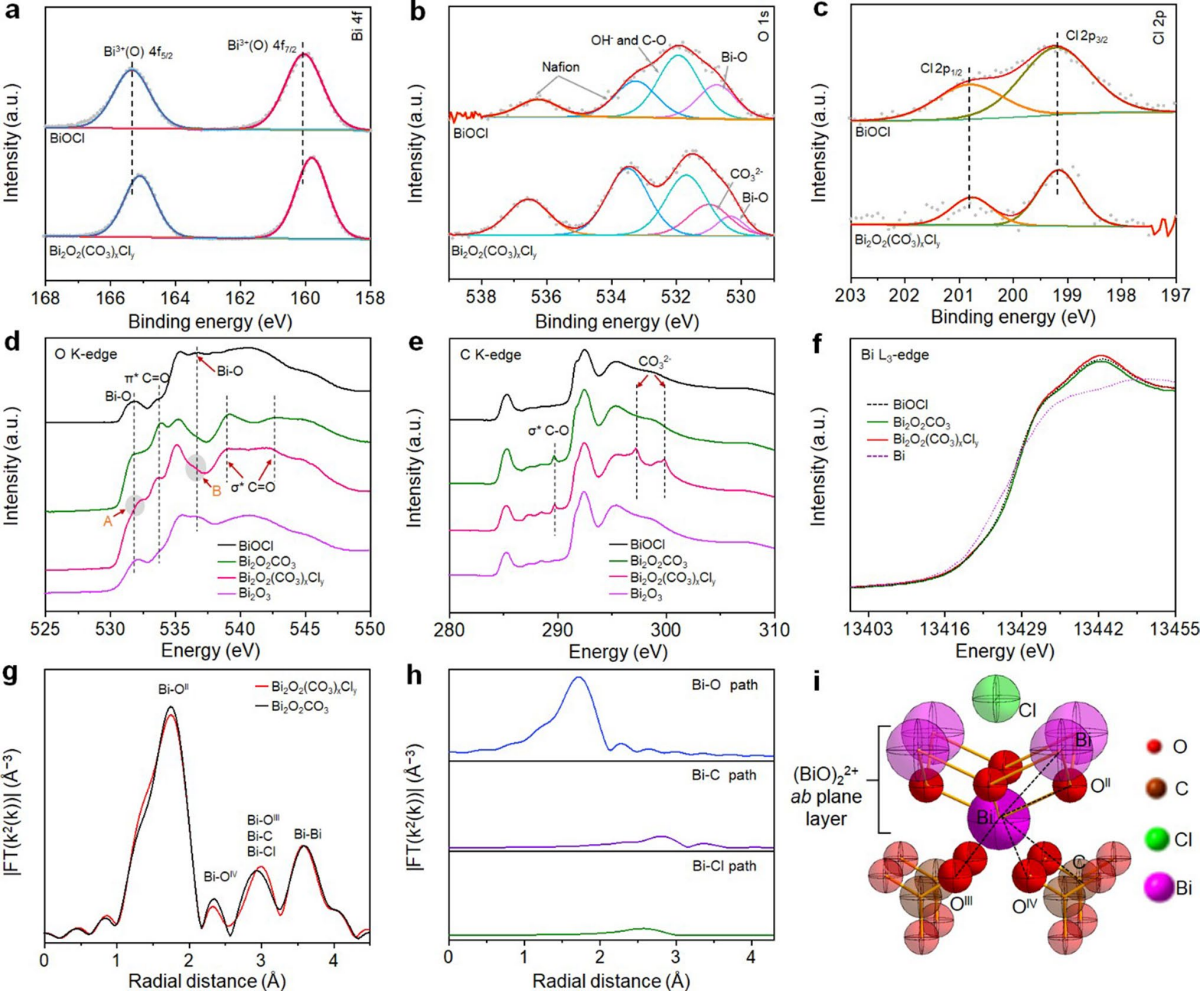
High-resolution XPS spectra of **a** Bi 4f, **b** O 1*s* and **c** Cl 1*s* obtained from the as-synthesized BiOCl-NSs and Bi_2_O_2_(CO_3_)_*x*_Cl_*y*_. **d** O K-edge spectra, **e** C K-edge spectra and **f** Bi L_3_-edge spectra of BiOCl, Bi_2_O_2_CO_3_, Bi_2_O_2_(CO_3_)_*x*_Cl_*y*_ and referenced samples. **g** Bi L_3_-edge *k*^*2*^-weighted FT-EXAFS spectra of Bi_2_O_2_(CO_3_)_*x*_Cl_*y*_ and Bi_2_O_2_CO_3_ in R space. **h** Fitting analysis of Bi_2_O_2_(CO_3_)_*x*_Cl_*y*_ using Bi–O, Bi–C and Bi–Cl paths. **i** Proposed geometric configuration of Bi_2_O_2_(CO_3_)_*x*_Cl_*y*_

The X-ray photoelectron spectroscopy (XPS) analysis was then carried out. The high-resolution XPS Bi 4f spectra (Fig. 2a) confirm the presence of Bi^3+^ and Bi–O bonds (160.0 and 165.3 eV) [31] in BiOCl. The Bi^3+^ peaks of Bi_2_O_2_(CO_3_)_*x*_Cl_*y*_ show a negative shift of 0.26 eV, consistent with that of reported Bi_2_O_2_CO_3_ [32]. Figure 2b shows the high-resolution XPS O 1*s* spectra of BiOCl and Bi_2_O_2_(CO_3_)_*x*_Cl_*y*_. The former could be deconvoluted into the binding energy peaks assignable to the Bi–O lattice O (530.8 eV), the surface adsorbed hydroxyl (~ 531.9 eV) and O species in Nafion (536.3, 533.3 and 531.9 eV) [33, 34], while the deconvoluted binding energy peaks at 530.2 and 531.0 eV from the later are ascribed to the Bi–O lattice O and C=O, respectively [35, 36]. The lattice O peaks in Bi_2_O_2_(CO_3_)_*x*_Cl_*y*_ shifted to lower energies due to the substitution of chloride by carbonate. The two binding energy peaks at 199.2 and 200.8 eV assignable to Cl 2*p*_3/2_ and Cl 2*p*_1/2_ can be deconvoluted from the high-resolution XPS Cl 2*p* spectra of both BiOCl and Bi_2_O_2_(CO_3_)_*x*_Cl_*y*_ (Fig. 2c), indicating the presence of the lattice Cl^−^ [37]. Notably, a Bi/Cl atomic ratio of 61.5:1 is determined from the XPS Cl 2*p* spectrum of Bi_2_O_2_(CO_3_)_*x*_Cl_*y*_ (Fig. S7), confirming the presence of chemically bonded Cl on the surface of Bi_2_O_2_(CO_3_)_*x*_Cl_*y*_.

The X-ray absorption spectroscopy (XAS) measurements were then conducted to probe the electronic structure and local atomic environments. The O K-edge near-edge X-ray absorption fine structure (NEXAFS) spectra of BiOCl, Bi_2_O_2_(CO_3_)_*x*_Cl_*y*_ and reference samples are shown in Fig. 2d. The observed binding energy peaks at 532.0 and 537.0 eV from Bi_2_O_2_(CO_3_)_*x*_Cl_*y*_ are assignable to the hybridization of O 2*p* with Bi 6* s* orbitals [38, 39], while the binding energy peak at 534 eV corresponds to the π^*^ C=O transition, indicating the presence of lattice carbonyl oxygen species [40]. The displayed binding energy peaks at 539.4 and 543.2 eV in the spectrum of Bi_2_O_2_(CO_3_)_*x*_Cl_*y*_ are ascribed to the non-equivalent σ^*^ C–O bonds in the carboxylic group originated from the adsorbed carbonate [41]. Based on the C K-edge NEXAFS spectra of Bi_2_O_2_(CO_3_)_*x*_Cl_*y*_ and reference samples (Fig. 2e), the binding energy peak at 289.5 eV can be attributed to the σ^*^ states of C–O [41], while the peaks at 297.2 and 299.8 eV are assignable to the σ^*^ C = O resonances associated with the presence of carbonate species [42]. It is to note that the O K-edge and C K-edge NEXAFS spectra obtained from Bi_2_O_2_CO_3_ and Bi_2_O_2_(CO_3_)_x_Cl_*y*_ exhibit very similar characteristics, implying that the crystal structure of Bi_2_O_2_CO_3_ in Bi_2_O_2_(CO_3_)_*x*_Cl_*y*_ is not noticeably altered by the presence of Cl^−^. According to the Bi L_3_-edge X-ray absorption near-edge structure (XANES) spectra (Fig. 2f), the same valence states of Bi^3+^ exist in BiOCl, Bi_2_O_2_CO_3_ and Bi_2_O_2_(CO_3_)_*x*_Cl_*y*_. Figure 2g shows the *k*^*2*^-weighted Fourier transformed Bi L_3_-edge extended X-ray absorption fine structure (*k*^*2*^-weighted FT-EXAFS) spectra of Bi_2_O_2_CO_3_ and Bi_2_O_2_(CO_3_)_*x*_Cl_*y*_. The peaks at 1.74, 2.33 and 3.58 Å assignable to the Bi–O and Bi–Bi bonds in the (BiO)_2_^2+^ *ab* plane are observed from both Bi_2_O_2_CO_3_ and Bi_2_O_2_(CO_3_)_*x*_Cl_*y*_, indicating an identical (BiO)_2_^2+^ *ab* plane in both Bi_2_O_2_CO_3_ and Bi_2_O_2_(CO_3_)_*x*_Cl_*y*_. The spectrum of Bi_2_O_2_CO_3_ shows a peak at 2.93 Å, corresponding to the interactions of Bi in (BiO)_2_^2+^ *ab* plane with the intercalated CO_3_^2−^ between the (BiO)_2_^2+^ *ab* plane layers (the interlayer). Notably, for Bi_2_O_2_(CO_3_)_*x*_Cl_*y*_, the peak is shifted to 3.01 Å, implying the differences in the interactions of Bi in (BiO)_2_^2+^ *ab* plane with the interlayer anions due to the presence of the intercalated Cl^−^ in the interlayer of Bi_2_O_2_(CO_3_)_*x*_Cl_*y*_. The fittings of the Bi L_3_-edge *k*^*2*^-weighted FT-EXAFS spectra of Bi_2_O_2_(CO_3_)_*x*_Cl_*y*_ and Bi_2_O_2_CO_3_ in *R* space and *k* space were then performed (Figs. 2h, S8 and Table S2) [43]. It unveils that the spectrum of Bi_2_O_2_(CO_3_)_*x*_Cl_*y*_ fits well with Bi_2_O_2_CO_3_ and Bi–Cl path, confirming the presence of the intercalated Cl^−^ in the interlayer. The coordination numbers (CNs) of Bi–O in the first coordination sphere of Bi_2_O_2_(CO_3_)_*x*_Cl_*y*_ and Bi_2_O_2_CO_3_ are 2.34 at 2.22 Å and 2.27 at 2.24 Å, respectively, further confirming an almost unchanged (BiO)_2_^2+^ *ab* plane coordination environment. For Bi_2_O_2_CO_3_, a Bi–C CN of 1.75 at 3.36 Å represents the interactions between the Bi atoms in the (BiO)_2_^2+^ *ab* plane and the intercalated CO_3_^2−^ in the interlayer. For Bi_2_O_2_(CO_3_)_*x*_Cl_*y*_, the measured Bi–C CN of 1.51 at 3.38 Å indicates a reduction in the Bi–C coordination. Notably, the fitting of the EXAFS spectrum of Bi_2_O_2_(CO_3_)_*x*_Cl_*y*_ using Bi–Cl backscattering path from the optimized structure corresponds to a Bi–Cl CN of 0.33, which is closely approximated to the dropped Bi–C CN, unambiguously confirming the presence of the intercalated Cl^−^ between (BiO)_2_^2+^ *ab* plane layers in Bi_2_O_2_(CO_3_)_*x*_Cl_*y*_. A likely Bi_2_O_2_(CO_3_)_*x*_Cl_*y*_ structure is shown in Fig. 2i. The above results confirm that under − 0.8 V (vs RHE) cathodic potential in CO_2_-saturated 0.5 M KHCO_3_ solution for 2 h, the BiOCl-NSs are electrochemically

### ***Operando*** Converting BiOCl into Bi_2_O_2_(CO_3_)_***x***_Cl_***y***_

**Fig. 3:**
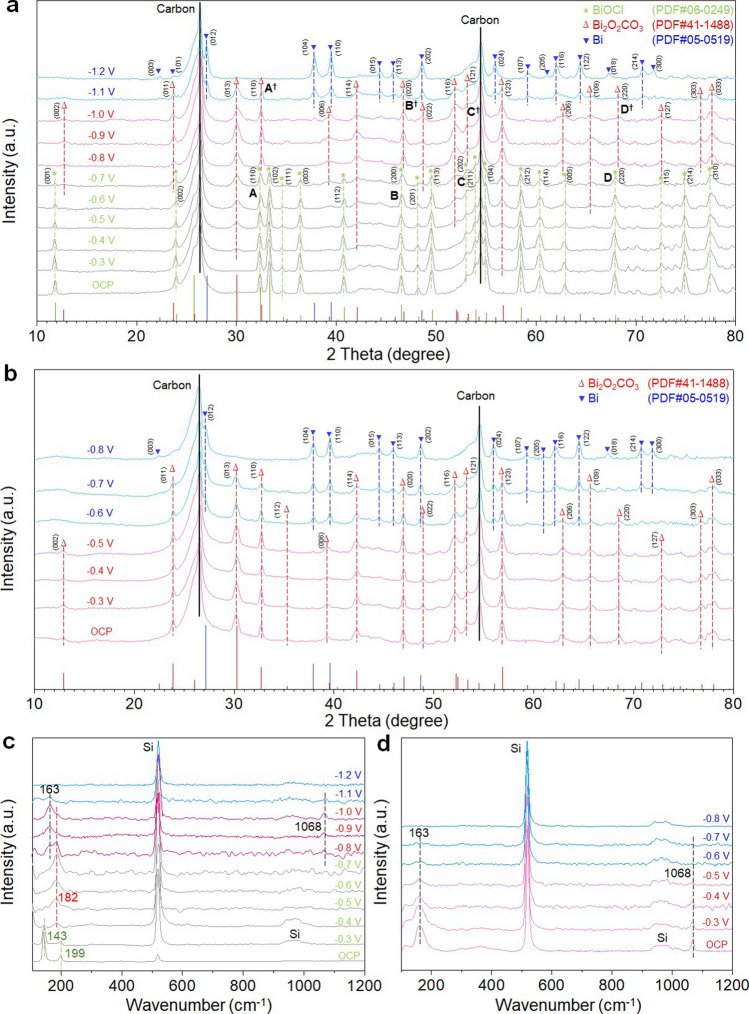
**a**,** b** *Operando* XRD patterns of the as-synthesized BiOCl-NSs and Bi_2_O_2_CO_3_ recorded from CO_2_-saturated 0.5 M KHCO_3_ solution under different cathodic potentials. **c**,** d** *Operando* Raman spectra of the as-synthesized BiOCl-NSs and Bi_2_O_2_CO_3_ recorded from CO_2_-saturated 0.5 M KHCO_3_ solution under different cathodic potentials

It is known that both of the tetragonal BiOCl and Bi_2_O_2_CO_3_ crystals (Fig. S9) belong to the Sillén crystal family, featuring a matlockite-type positively charged (BiO)_2_^2+^ *ab* plane layer structure stacking between the negatively charged bichloride and “standing-on-end” CO_3_^2−^ anions slabs, respectively. Comparing to the *d* spacing of {001} faceted BiOCl along the *c* axis (7.83 Å), the *d spacing* of {002} faceted Bi_2_O_2_CO_3_ is markedly reduced to 6.84 Å. Therefore, under apt cathodic potentials, due to the layer structure similarity, and the apparently decreased *d* spacing of {002} faceted Bi_2_O_2_CO_3_, the transformation of BiOCl to Bi_2_O_2_CO_3_ could occur via the intercalative substitution of the interlayer Cl^−^ with CO_3_^2−^ through a glide of the neighboring (BiO)_2_^2+^ *ab* planes along [100] and [010] directions with the translational distances of ½ *a* and ½ *b*, respectively [17]. To depict the structural evolution processes under the *operando* CO_2_RR conditions, the BiOCl-NSs immobilized on the carbon fiber paper were subjected to different cathodic potentials (*E*_App_) in CO_2_-saturated 0.5 M KHCO_3_ solution, and the XRD patterns were *operando* recorded. The XRD patterns recorded under the open circuit potential (OCP) and *E*_App_ ≤ − 0.2 V (Fig. 3a) are almost identical to that of the as-synthesized BiOCl (Fig. S1). The initial conversion of BiOCl to Bi_2_O_2_(CO_3_)_*x*_Cl_*y*_ occurs at *E*_App_ = − 0.3 V as indicated by the observed diffraction peak at 56.9° corresponding to {123} faceted Bi_2_O_2_CO_3_. When *E*_App_ is increased from − 0.3 to − 0.7 V, although the recorded XRD patterns are still dominated by the diffraction patterns of BiOCl, the progress of converting BiOCl to Bi_2_O_2_(CO_3_)_*x*_Cl_*y*_ is evidenced by the progressively increased intensities of Bi_2_O_2_CO_3_ diffraction peaks and the accompanied decrease in the intensities of BiOCl diffraction peaks. With *E*_App_ = − 0.8 V, all recorded diffraction peaks belong to Bi_2_O_2_CO_3_ (PDF No. 41-1488), signifying the complete conversion of BiOCl to Bi_2_O_2_(CO_3_)_*x*_Cl_*y*_. The structural evolution and the time required to completely convert BiOCl to Bi_2_O_2_(CO_3_)_*x*_Cl_*y*_ under *E*_App_ = − 0.8 V were subsequently investigated (Fig. S10). As can be seen, BiOCl is fully covered to Bi_2_O_2_(CO_3_)_*x*_Cl_*y*_ within 60 min under *E*_App_ = − 0.8 V. As disclosed in Fig. 3a, Bi_2_O_2_(CO_3_)_*x*_Cl_*y*_ remains as the sole product when − 0.8 V ≤ *E*_App_ ≤ − 1.0 V. With *E*_App_ = − 1.1 V, the bismuth in Bi_2_O_2_(CO_3_)_*x*_Cl_*y*_ is partially reduced to the metallic phased Bi^o^ as evidenced by the appearance of the diffraction peaks assignable to rhombohedral phased Bi^o^ (PDF No. 05–0519). With *E*_App_ = − 1.2 V, all of the recorded diffraction peaks belong to the rhombohedral phased Bi^o^, confirming the ultimate conversion of Bi_2_O_2_(CO_3_)_*x*_Cl_*y*_ to the metallic Bi^o^ phase. As shown in Fig. S11, the BiOCl-derived Bi^o^ at *E*_App_ = − 1.2 V is formed by the aggregated Bi^o^ NSs with the exposed {001} facets. Notably, the required cathodic potential to convert Bi_2_O_2_(CO_3_)_*x*_Cl_*y*_ to Bi^o^ is more negative than those reported potentials to reduce Bi_2_O_2_CO_3_ to Bi^o^ [44], {Lv, 2017 #1765}inferring a superior electrochemical stability of Bi_2_O_2_(CO_3_)_x_Cl_*y*_ over Bi_2_O_2_CO_3_, which might be attributed to the presence of Cl^−^ in Bi_2_O_2_(CO_3_)_*x*_Cl_*y*_. To confirm this, the pure tetragonal phased Bi_2_O_2_CO_3_ nanosheets (Figs. S12–S14) were synthesized by a wet-chemistry method [43] and subjected to different cathodic potentials in CO_2_-saturated 0.5 M KHCO_3_ solution. Figure 3b shows the *operando* recorded XRD patterns. The formation Bi^o^ occurs at *E*_App_ = − 0.6 V, while the Bi_2_O_2_CO_3_ is fully converted to the rhombohedral phased metallic Bi^o^ (PDF No. 05-0519) at *E*_App_ = − 0.8 V, confirming that the presence of Cl^−^ in Bi_2_O_2_(CO_3_)_*x*_Cl_*y*_ is responsible for the improved electrochemical stability. It is noteworthy that compared to the characteristic diffraction peaks of Bi_2_O_2_CO_3_, all of the recorded characteristic diffraction peaks from Bi_2_O_2_(CO_3_)_*x*_Cl_*y*_ are shifted slightly toward lower angles (Fig. S15), indicating an expended *d* spacing in Bi_2_O_2_(CO_3_)_*x*_Cl_*y*_ due to the presence of Cl^−^ in the interlayer. The above *operando* XRD studies unveil that the electrochemical conversion of BiOCl to Bi_2_O_2_(CO_3_)_*x*_Cl_*y*_ is realized by the cathodic potential-promoted anion-exchange in the interlayer between the (BiO)_2_^2+^ *ab* planes.

To further elaborate the electrochemical conversion pathway, the *operando* potential-dependent Raman spectra of BiOCl immobilized on the carbon fiber paper were recorded under different cathodic potentials in CO_2_-saturated 0.5 M KHCO_3_ solution (Fig. 3c). Under the OCP and *E*_App_ ≤ − 0.3 V conditions, the recorded spectra are almost identical to that of the as-synthesized BiOCl-NSs (Fig. S2). With *E*_App_ = − 0.4 V, the characteristic peaks of BiOCl at 143 ($A_{1g}^{1}$) and 199 cm^−1^ ($A_{1g}^{2}$) are markedly reduced and disappeared, respectively, which are accompanied by the appearance of a new peak at 182 cm^−1^ that might be assigned to the *A*_*1g*_ mode of the intercalated Cl^−^ in the interlayer, although this could be complicated by the reduced crystal symmetry due to the disorder or free rotation of CO_3_^2−^ in the interlayer [29, 30]. These observed changes in the Raman spectrum signify the initial conversion of BiOCl to Bi_2_O_2_(CO_3_)_*x*_Cl_*y*_ at *E*_App_ = − 0.4 V, consistent with the *operando* XRD observation shown in Fig. 3a. At *E*_App_ = − 0.8 V, two new peaks at 163 and 1068 cm^−1^ attributed to the external vibration of Bi_2_O_2_CO_3_ crystal and the *ν*_1_ mode of CO_3_^2−^ anions between the (BiO)_2_^2+^ planes appear, signifying the complete conversion of BiOCl to Bi_2_O_2_(CO_3_)_*x*_Cl_*y*_. Within − 0.8 V ≤ *E*_App_ ≤ − 1.0 V, the peak at 182 cm^−1^ (*A*_*1g*_ mode) is rapidly decreased, while the peaks at 163 and 1068 cm^−1^ are evolved and intensified, which are likely due to the changes in the local atomic symmetry rather than the crystal cell parameters because of the unchanged *operando* XRD patterns within the same potential range (Fig. 3a). It is known that the high-intensity Raman bands between 150 and 200 cm^−1^ normally correspond to the out-of-plane lattice vibrations of the Bi atoms perpendicular to the (BiO)_2_^2+^ layers [45]. Because the phonon frequencies are sensitive to the dopant-induced asymmetry and the interlayer thickness, therefore, the blueshifted phonons from 182 to 163 cm^−1^ could be resulted from the suppressed vibrations of the (BiO)_2_^2+^ layers along *c* direction due to the replaced Cl^−^ by CO_3_^2−^ at relatively high cathodic potentials. The characteristic Raman peaks of Bi_2_O_2_CO_3_ at 163 and 1068 cm^−1^ are dramatically decreased under *E*_App_ = − 1.1 V and vanished at *E*_App_ = − 1.2 V due to the formation of metallic Bi^o^, consistent with the *operando* XRD observations. For comparative purpose, the *operando* potential-dependent Raman spectra of the pure tetragonal phased Bi_2_O_2_CO_3_ nanosheets were obtained (Fig. 3d). When *E*_App_ ≤ − 0.5 V, the peak at 182 cm^−1^ associating with the *A*_1g_ mode of the intercalated Cl^−^ in the CO_3_^2−^ slab is absent, while the characteristic Raman peaks of Bi_2_O_2_CO_3_ at 163 and 1068 cm^−1^ are apparent, however, rapidly extinct when *E*_App_ ≥− 0.6 V due to the formation of metallic Bi^o^, consistent with the *operando* XRD observations shown in Fig. 3b. This further confirms that compared to the chloride-free Bi_2_O_2_CO_3_, the reduction of Bi_2_O_2_(CO_3_)_*x*_Cl_*y*_ to metallic Bi^o^ requires a much higher cathodic potential due to presence of the intercalated Cl^−^ in the CO_3_^2−^ slab, signifying a noticeably improved electrochemical stability. These *operando* Roman studies further suggest that the conversion of BiOCl to Bi_2_O_2_(CO_3_)_*x*_Cl_*y*_ is achieved by the cathodic potential-promoted anion-exchange in the interlayer.

### CO_2_RR Performance

**Fig. 4:**
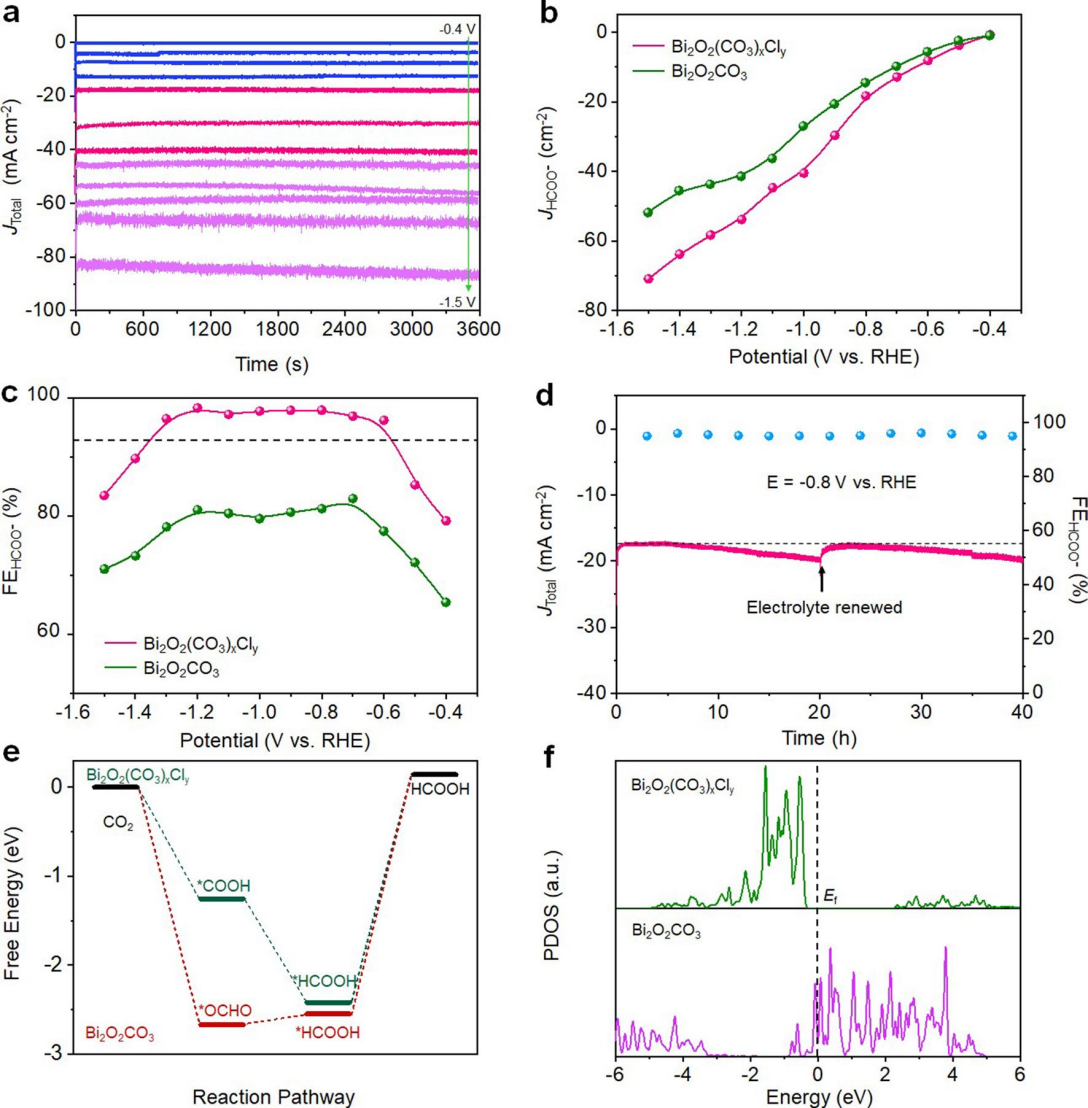
**a** Chronoamperometric curves of Bi_2_O_2_(CO_3_)_*x*_Cl_*y*_ recorded from CO_2_-saturated 0.5 M KHCO_3_ solution under different cathodic potentials. **b**,** c** Plots of HCOOH partial current density and Faradic efficiency against catholic potential for Bi_2_O_2_(CO_3_)_*x*_Cl_*y*_- and Bi_2_O_2_CO_3_-catalyzed CO_2_RR. **d** Chronoamperometric curves and FE_HCOOH_ of Bi_2_O_2_(CO_3_)_*x*_Cl_*y*_ at − 0.8 V versus RHE. **e** Free energy diagrams of Bi_2_O_2_(CO_3_)_*x*_Cl_*y*_- and Bi_2_O_2_CO_3_-catalyzed CO_2_ reduction to HCOOH. **f** PDOSs plots of Bi_2_O_2_(CO_3_)_*x*_Cl_*y*_- and Bi_2_O_2_CO_3_-catalyzed CO_2_ reduction to HCOOH

All electrochemical measurements were performed using a three-electrode electrochemical system with Bi_2_O_2_(CO_3_)_*x*_Cl_*y*_ or Bi_2_O_2_CO_3_ working electrode in CO_2_- or Ar-saturated 0.5 M KHCO_3_ solution. When *E*_App_ > − 0.5 V (vs EHE), the linear sweep voltammetry (LSV) responses of Bi_2_O_2_(CO_3_)_*x*_Cl_*y*_ in CO_2_-saturated solution display higher cathodic current densities than that obtained from the Ar-saturated solution (Fig. S16), indicating a superior electrocatalytic activity of Bi_2_O_2_(CO_3_)_*x*_Cl_*y*_ toward CO_2_RR. The potentiostatic experiments were then performed under different cathodic potentials to examine the electrocatalytic CO_2_RR activity and selectivity. Figure 4a shows the chronoamperometric curves of Bi_2_O_2_(CO_3_)_*x*_Cl_*y*_ from the CO_2_-saturated 0.5 M KHCO_3_ solution. The reaction products in gaseous and aqueous phases were qualitatively identified and quantitatively determined by the gas chromatography (GC) and nuclear magnetic resonance (NMR). For all cases investigated, H_2_ is identified as the sole product in the gaseous phase, while the formate is found to be the sole product in the aqueous phase. The NMR determined formate concentrations (Figs. S17 and S18) corresponding to the chronoamperometric curves shown in Fig. 4a were used to calculate the corresponding FE_HCOO_- and *J*_HCOO_- values. Figure 4b shows the plot of *J*_HCOO_- against *E*_App_. For both Bi_2_O_2_(CO_3_)_*x*_Cl_*y*_ and Bi_2_O_2_CO_3_, an increase in *E*_App_ leads to an increase in *J*_HCOO_-. For a given *E*_App_, the observed *J*_HCOO_- from Bi_2_O_2_(CO_3_)_*x*_Cl_*y*_ is higher than that observed from Bi_2_O_2_CO_3_, implying a superior CO_2_RR activity of Bi_2_O_2_(CO_3_)_*x*_Cl_*y*_ over Bi_2_O_2_CO_3_. At *E*_App_ = − 0.8 V, the *J*_HCOO_- attained by Bi_2_O_2_(CO_3_)_*x*_Cl_*y*_ is 18.4 mA cm^−2^, higher than that of Bi_2_O_2_CO_3_ (14.2 mA cm^−2^). Figure 4c shows the plot of FE_HCOO_- (derived from Fig. 4a) against *E*_App_. For Bi_2_O_2_(CO_3_)_*x*_Cl_*y*_, an increase in *E*_App_ from − 0.4 to − 0.6 V leads to a rapidly increased FE_HCOO_- from 79.2 to 96.2%, and further increasing *E*_App_ to − 0.8 V leads to an increased FE_HCOO_- to 97.9%. FE_HCOO_- remains almost unchanged when *E*_App_ is further increased to − 1.0 V, while the corresponding *J*_HCOO_- is increased to 40.5 mA cm^−2^ (Fig. 4b). Based on the *operando* XRD and Raman observations (Fig. 3a, c), the formation of the metallic phased Bi^o^ will not occur with *E*_App_ ≤ − 1.0 V, therefore, the observed changes in FE_HCOO_- from the potential range of − 0.4 V ≤ *E*_App_ ≤ − 1.0 V reflect the influence of potential on CO_2_RR selectivity of Bi_2_O_2_(CO_3_)_*x*_Cl_*y*_. Although Bi_2_O_2_(CO_3_)_*x*_Cl_*y*_ is partially converted to metallic Bi–NSs within − 1.0 V ≤ *E*_App_ ≤ − 1.2 V (Fig. 3a, c), the high FE_HCOO_- can still be attained due to the electrocatalytic activity of Bi^o^ toward CO_2_RR under high cathodic potentials [17]. When *E*_App_ > − 1.2 V, the observed decrease in FE_HCOO_- is due to the intensified competition from HER [7]. Interestingly, for pure Bi_2_O_2_CO_3_, a rapidly increased FE_HCOO_- from 65.5 to 77.5% is observed when *E*_App_ is increased from − 0.4 to − 0.6 V and reached a maxima FE_HCOO_- of 83.0% at *E*_App_ = − 0.7 V, where Bi_2_O_2_CO_3_ is partially converted to the metallic Bi^o^. With − 0.8 V ≤ *E*_App_ ≤ − 1.2 V, Bi_2_O_2_CO_3_ is fully converted to the metallic Bi^o^ and the slightly decreased FE_HCOO_- reflects the influence of potential on CO_2_RR selectivity of metallic Bi^o^ rather than that of Bi_2_O_2_CO_3_. When *E*_App_ > − 1.2 V, FE_HCOO_- is rapidly decreased due to the intensified competition from HER.

The chronoamperometric stability of Bi_2_O_2_(CO_3_)_*x*_Cl_*y*_ (Fig. 4d) was evaluated over a 20 h period in CO_2_-saturated 0.5 M KHCO_3_ solution at *E*_App_ = − 0.8 V vs RHE. While FE_HCOO_- of ~ 95% is well retained, a 12.1% increase in the cathodic current density is observed, indicating an increased *J*_HCOO_-. Interestingly, when the used electrolyte is replaced by the fresh one, an almost identical chronoamperometric curve is obtainable with ~ 12% increase in the cathodic current density at 20 h, indicating an excellent long-term stability of Bi_2_O_2_(CO_3_)_*x*_Cl_*y*_ [46]. The excellent electrocatalytic stability of Bi_2_O_2_(CO_3_)_*x*_Cl_*y*_ can be attributed to its excellent structural stability as evidenced by the almost unchanged XRD pattern (Fig. S19), Raman spectra (Fig. S20), as well as SEM and TEM images (Fig. S21) of Bi_2_O_2_(CO_3_)_*x*_Cl_*y*_ after the chronoamperometric stability test. The chronoamperometric stability of Bi_2_O_2_CO_3_ was also evaluated at *E*_App_ = − 0.8 V vs RHE (Fig. S22). Over a 20 h testing period, FE_HCOO_- is decreased from 86.2 to 80.0%, while the cathodic current density is increased from 12.5 to 14.2 mA cm^−2^. However, after the chronoamperometric stability test, the pure tetragonal phased Bi_2_O_2_CO_3_ is fully converted to the metallic phased Bi^o^ (Figs. S23–S26). The stability test result confirms that Bi_2_O_2_(CO_3_)_*x*_Cl_*y*_ fabricated under *operando* CO_2_RR conditions possesses excellent stability.

### DFT Calculations

It is known that electrocatalytic CO_2_RR to HCOOH has normally proceeded via a proton-coupled electron transfer (PCET) step to form ^*^COOH or ^*^OCHO intermediates and followed by another PCET step to generate HCOOH [47]. It is also known that the CO_2_RR pathway depends strongly on the adsorption energy of the intermediates [48]. DFT calculations were therefore carried out to determine the preferential intermediates of the pure tetragonal phased Bi_2_O_2_CO_3_- and Bi_2_O_2_(CO_3_)_*x*_Cl_*y*_-catalyzed CO_2_ reduction to HCOOH. Our DFT calculations unveil that ^*^OCHO intermediate can preferentially adsorb on the {001} faceted Cl-free Bi_2_O_2_CO_3_ surface [49] with an adsorption free energy (ΔG_*OCHO_) of − 2.67 eV (Fig. S27a), while no stable structure of ^*^COOH intermediate adsorbed on the {001} faceted Cl-free Bi_2_O_2_CO_3_ can be obtained. These results imply that the pure tetragonal phased Bi_2_O_2_CO_3_-catalyzed CO_2_ reduction to HCOOH has proceeded via a ^*^OCHO intermediate pathway. In contrast, our initial DFT calculations are failed to obtain a stable structure of ^*^OCHO intermediate adsorbed on Bi_2_O_2_(CO_3_)_*x*_Cl_*y*_ surface. Nonetheless, further DFT calculations unveil that the ^*^COOH intermediate is apt to absorb to the Bi_2_O_2_(CO_3_)_*x*_Cl_*y*_ surface with a ΔG_*COOH_ of − 1.25 eV (Fig. S27b), inferring that the Bi_2_O_2_(CO_3_)_*x*_Cl_*y*_-catalyzed CO_2_ reduction to HCOOH has proceeded via a ^*^COOH intermediate pathway. Figure 4e illustrates the free energy diagrams of Bi_2_O_2_CO_3_- and Bi_2_O_2_(CO_3_)_*x*_Cl_*y*_-catalyzed CO_2_ reduction to HCOOH. During the 1st PCET step, the formation of ^*^OCHO on Bi_2_O_2_CO_3_ surface and ^*^COOH on Bi_2_O_2_(CO_3_)_*x*_Cl_*y*_ surface is exothermic. During the 2nd PCET step, the formation of ^*^HCOOH on Bi_2_O_2_(CO_3_)_*x*_Cl_*y*_ is exothermic, while on Bi_2_O_2_CO_3_ is endothermic. The desorption of ^*^HCOOH from Bi_2_O_2_CO_3_ and Bi_2_O_2_(CO_3_)_*x*_Cl_*y*_ to form HCOOH are energetically uphill. However, the desorption of ^*^HCOOH from Bi_2_O_2_(CO_3_)_*x*_Cl_*y*_ requires 2.56 eV to proceed, which is 0.13 eV lower than that of Bi_2_O_2_CO_3_ (2.69 eV), indicating a better kinetic activity of Bi_2_O_2_(CO_3_)_*x*_Cl_*y*_ over the Cl-free Bi_2_O_2_CO_3_. The poor kinetic activity of Bi_2_O_2_CO_3_ could be resulted from the excessively high adsorption energy of ^*^OCHO impeded active sites turnover. Figure 4f shows the projected density of states (PDOS) of the *p* bands of Bi sites in Bi_2_O_2_(CO_3_)_*x*_Cl_*y*_ and the {001} faceted Bi_2_O_2_CO_3_. As can be seen, the PDOS of the {001} faceted Bi_2_O_2_CO_3_ near Fermi level is dominated by the *p*-orbital electron states with much higher electronic densities than that of Bi_2_O_2_(CO_3_)_*x*_Cl_*y*_, indicating a higher reactivity for ^*^OCHO intermediate adsorption, hence an impeded active sites regeneration [50, 51]. In addition, the high PDOS density of the {001} faceted Bi_2_O_2_CO_3_ near Fermi level indicates high densities of the unoccupied *p*-orbital states of Bi in the {001} faceted Bi_2_O_2_CO_3_ with an estimated lowest unoccupied molecular orbital (LUMO) energy of 0.4 eV above Fermi level, corresponding to a LUMO potential of − 0.4 eV. In strong contrast, for Bi_2_O_2_(CO_3_)_*x*_Cl_*y*_, the *p*-orbital electron states can only be observed at 2.3 eV above the Fermi level with low densities, indicating low unoccupied *p*-orbital states of Bi in Bi_2_O_2_(CO_3_)_*x*_Cl_*y*_, corresponding to a LUMO potential of − 2.3 eV. It is known that for semiconductor electrodes, the reduction reaction takes place via the injection of electrons into LUMO. Therefore, the LUMO potential corresponds to the minimum required cathodic potential for electron injection. As illustrated in Fig. S28, compared to Bi_2_O_2_CO_3_, the higher LUMO potential of Bi_2_O_2_(CO_3_)_*x*_Cl_*y*_ infers that a higher cathodic potential is required to convert Bi^3+^ in Bi_2_O_2_(CO_3_)_*x*_Cl_*y*_ to metallic Bi^o^, which explains the superior electrochemical stability of Bi_2_O_2_(CO_3_)_*x*_Cl_*y*_ over Bi_2_O_2_CO_3_ under CO_2_RR conditions.

## Conclusions

In summary, we reported an approach to electrochemically convert bismuth oxychloride (BiOCl) into chloride-containing bismuth subcarbonate (Bi_2_O_2_(CO_3_)_*x*_Cl_*y*_) under *operando* CO_2_RR conditions. We demonstrated that the *operando* synthesis is an effective strategy to enhance the electrochemical stability of bismuth-based electrocatalysts. The exemplified approach in this work could be widely applicable to enhance the electrochemical stabilities of other electrocatalysts for other reactions.

## Supplementary Information

Below is the link to the electronic supplementary material.

Supplementary file1 (PDF 3927 KB)
