# Supplementary material for: Operando Converting BiOCl into Bi2O2(CO3)xCly for Efficient Electrocatalytic Reduction of Carbon Dioxide to Formate
Source: Nanomicro Lett. 2022 May 3;14:121. doi: 10.1007/s40820-022-00862-0 (PMC9065225; doi:10.1007/s40820-022-00862-0)
Supplement: Supplementary file 1 — Supplementary file1 (PDF 3927 KB) [file 40820_2022_862_MOESM1_ESM.pdf]

Supporting Information for

Operando Converting BiOCl into $Bi_2O_2(CO_3)_xCl_y$ for Efficient Electrocatalytic Reduction of Carbon Dioxide to Formate

Huai Qin Fu[1], Junxian Liu[1], Nicholas M. Bedford[2], Yun Wang[1], Joshua Wright[3], Peng Fei Liu[4], Chun Fang Wen[4], Liang Wang[1], Huajie Yin[1], Dongchen Qi[5], Porun Liu[1, *], Hua Gui Yang[4], and Huijun Zhao[1, *]

[1]Centre for Catalysis and Clean Energy, Gold Coast Campus, Griffith University, Gold Coast, Queensland 4222, Australia

[2]School of Chemical Engineering, University of New South Wales, Sydney, NSW 2052, Australia

[3]Department of Physics, Illinois Institute of Technology, Chicago, Illinois, 60616, United States

[4]Key Laboratory for Ultrafine Materials of Ministry of Education, School of Materials Science and Engineering, East China University of Science and Technology, Shanghai 200237, P. R. China

[5]Centre for Materials Science, School of Chemistry and Physics, Queensland University of Technology, Brisbane, Queensland 4001, Australia

*Corresponding authors. E-mail: p.liu@griffith.edu.au (Porun Liu), h.zhao@griffith.edu.au (Huijun Zhao)

Supplementary Figures and Tables

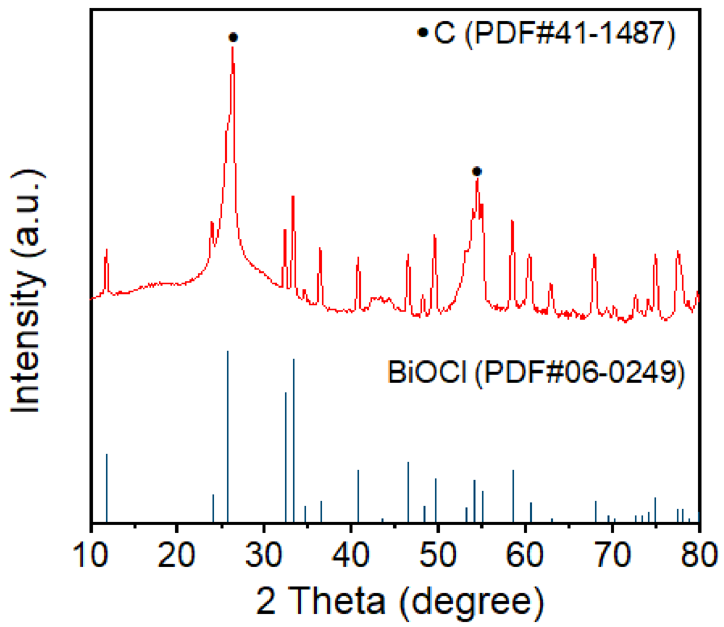

Fig. S1 XRD pattern of the as-synthesized BiOCl-NSs

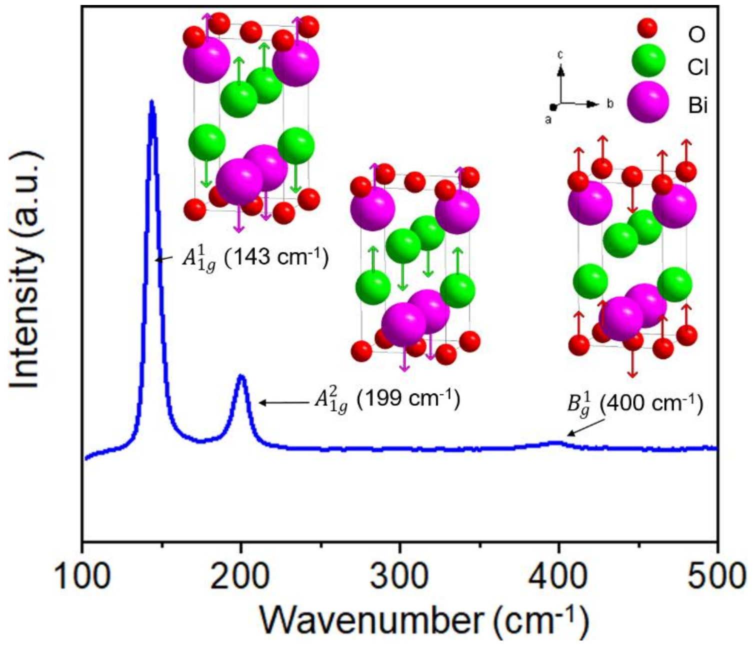

Fig. S2 Raman spectra of the as-synthesized BiOCl-NSs

The BiOCl crystal possesses a tetragonal structure (space group D^7_{4h}-P4/nmm (129), Z = 2). The Bi^{3+} site has C_{4v} symmetry. The primitive cell consists of 6 monoatomic sites. The structure of the unreducible representation of the 15 normal modes of vibrations is: $\Gamma = 2\ A_{1g} + 2\ A_{2u} + B_{1g} + 3\ E_g + 2\ E_u$, in which the A^1_{1g} (143 cm^{-1}), A^2_{1g} (199 cm^{-1}) and B^1_g (400 cm^{-1}) species are resolved in the data while A_{2u} and E_u are IR active [S1].

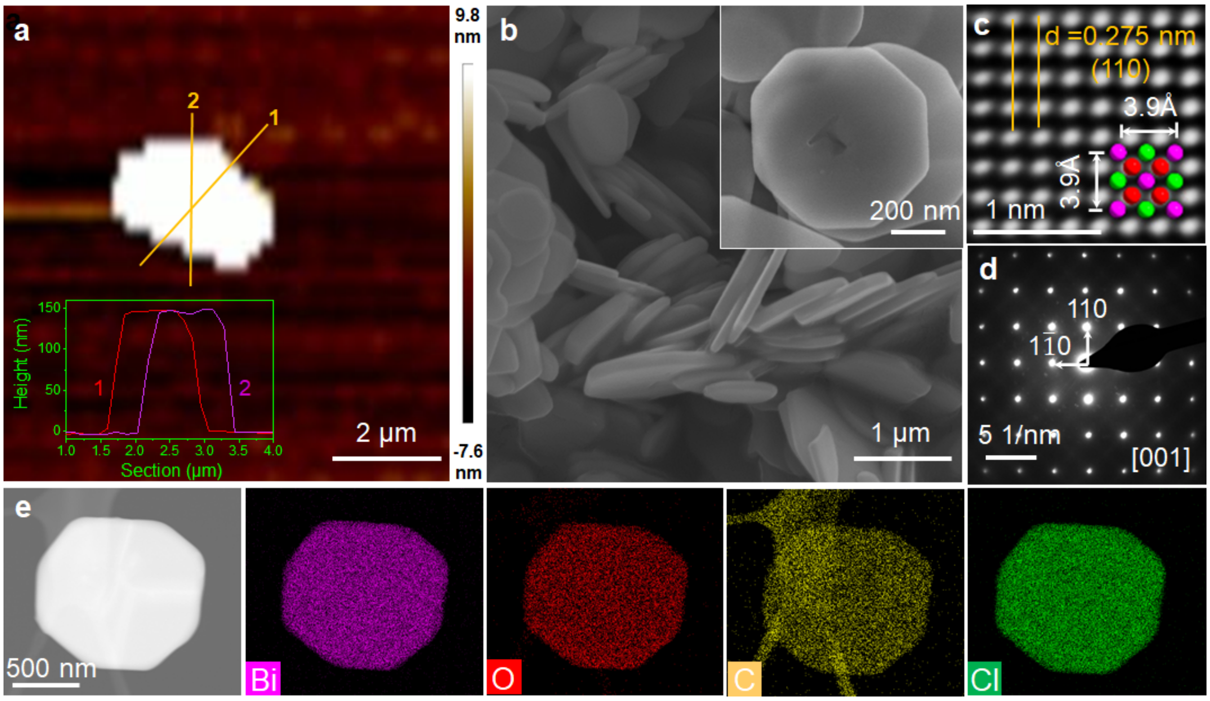

Fig. S3 (**a**) AFM image and corresponding height profile, (**b**) FESEM images, (**c**) high resolution IFFT-TEM image, (**d**) corresponding SAED pattern, (**e**) HAADF-STEM image and corresponding element mapping images of BiOCl-NSs

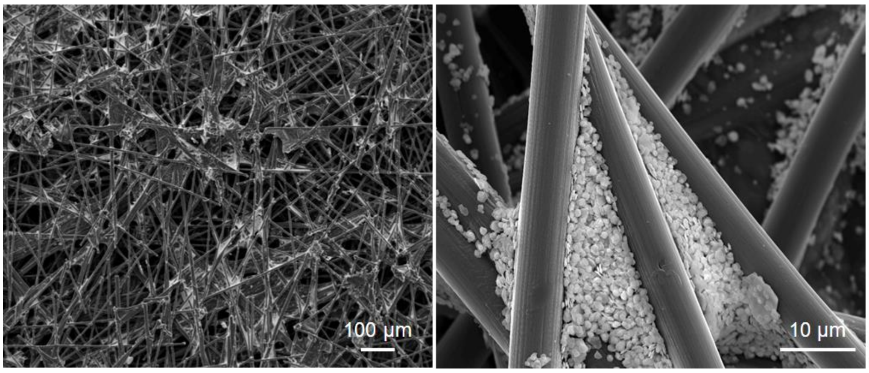

Fig. S4 FESEM images of BiOCl-NSs loaded on the carbon paper substrate

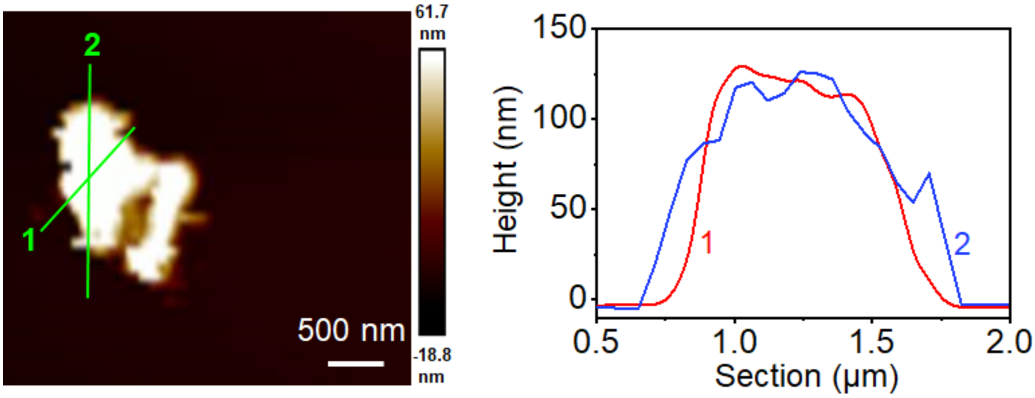

Fig. S5 AFM image and corresponding height profile of $Bi_2O_2(CO_3)_xCl_y$

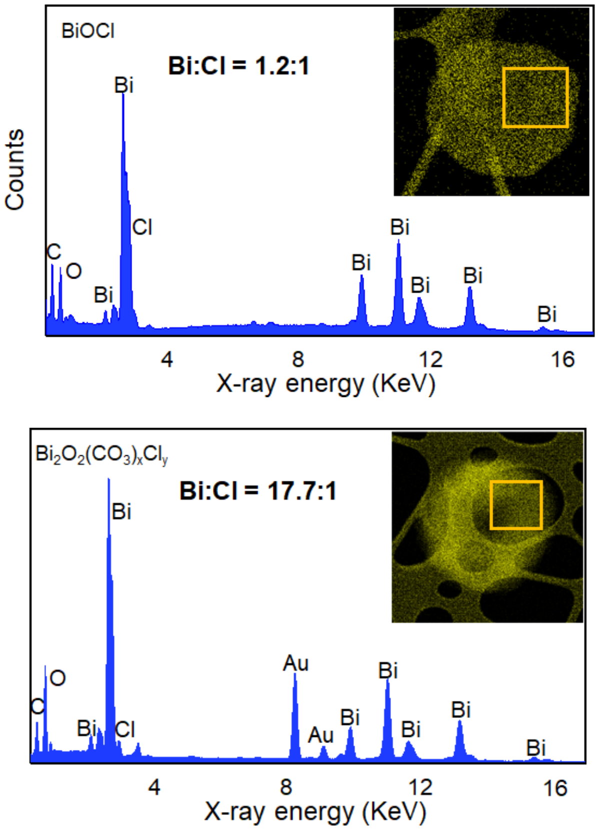

Fig. S6 STEM-EDX spectra of BiOCl-NSs and $Bi_2O_2(CO_3)_xCl_y$ obtained from the selected area

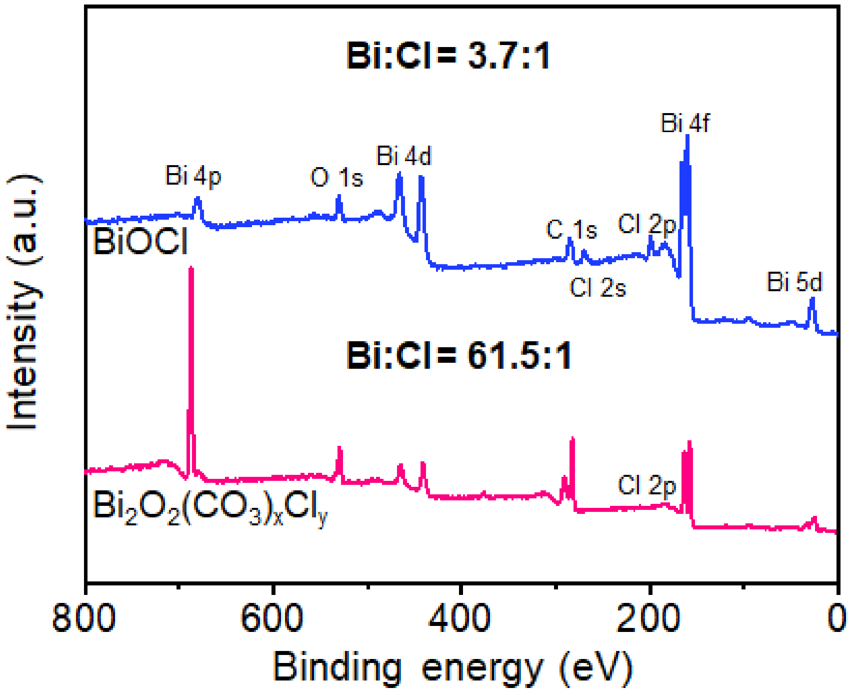

Fig. S7 Survey XPS spectra of BiOCl-NSs and $Bi_2O_2(CO_3)_xCl_y$

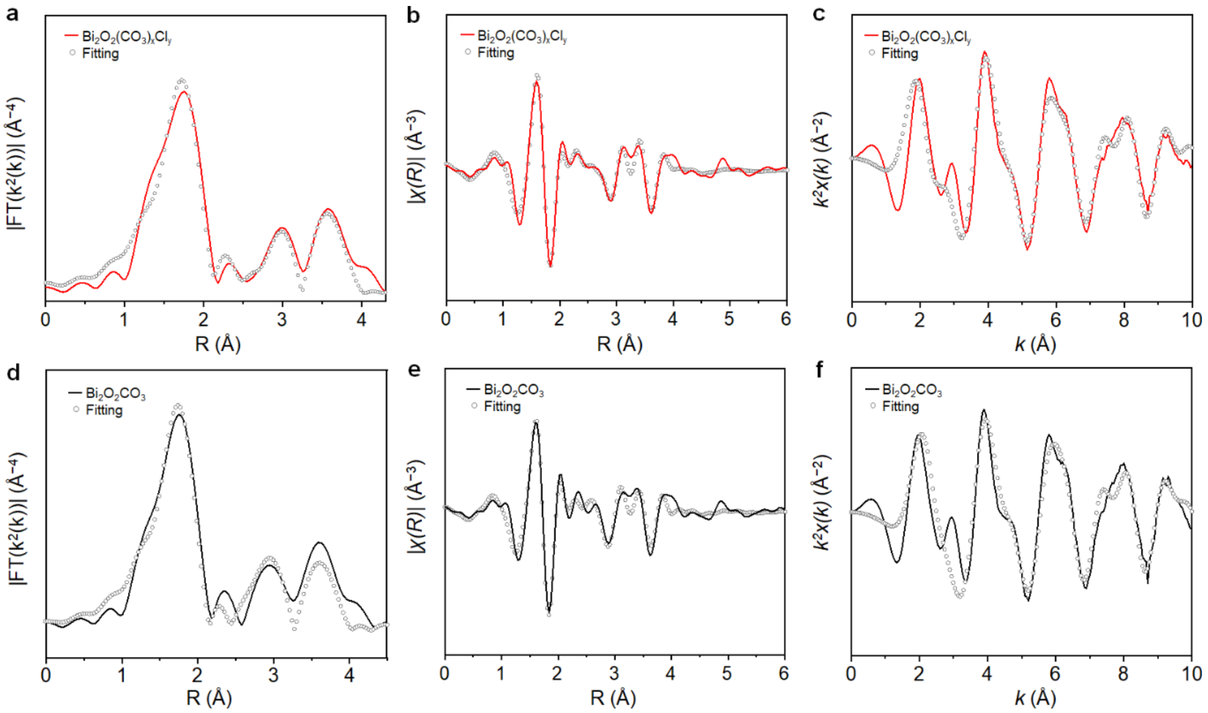

Fig. S8 (**a**) Bi L_3-edge k^2-weighted FT-EXAFS spectrum and fitting curve of $Bi_2O_2(CO_3)_xCl_y$ in R space, and (**b**, **c**) Magnitude parts of Bi L_3-edge spectra and corresponding fitting curves of $Bi_2O_2(CO_3)_xCl_y$ in R space and K space, respectively. (**d**) Bi L_3-edge k^2-weighted FT-EXAFS spectrum and fitting curve of $Bi_2O_2CO_3$ in R space, and (**e**, **f**) Magnitude parts of Bi L_3-edge spectra and corresponding fitting curves of $Bi_2O_2CO_3$ in R space and K space, respectively

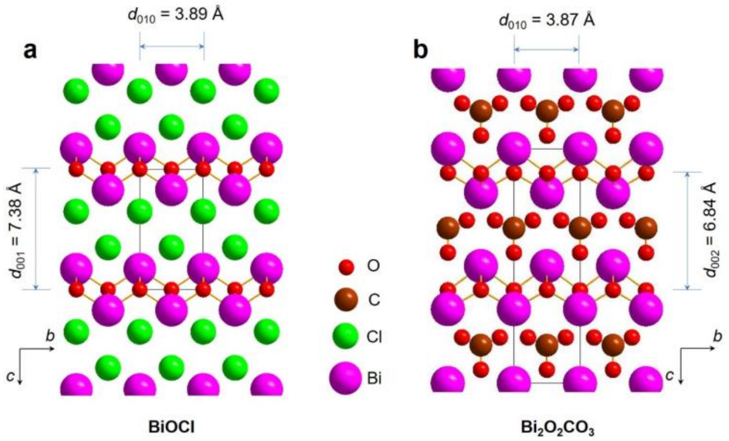

Fig. S9 Schematic diagram of (**a**) BiOCl and (**b**) $Bi_2O_2CO_3$ crystal structure viewed from *a* axis. The black lines represent the edges of a unit cell. Red, brown, green and purple spheres represent O, C, Cl and Bi atoms, respectively

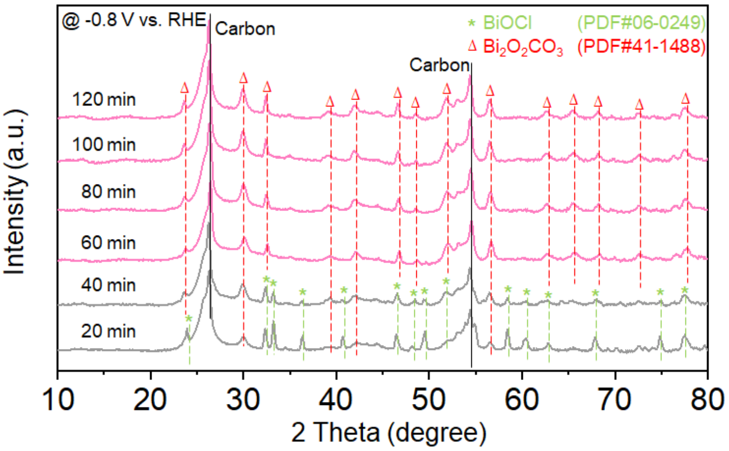

Fig. S10 *Operando* time-dependent XRD patterns recorded during electrochemical conversion of BiOCl-NSs to $Bi_2O_2(CO_3)_xCl_y$ at -0.8 V (vs RHE) in CO_2-saturated 0.5 M $KHCO_3$ solution

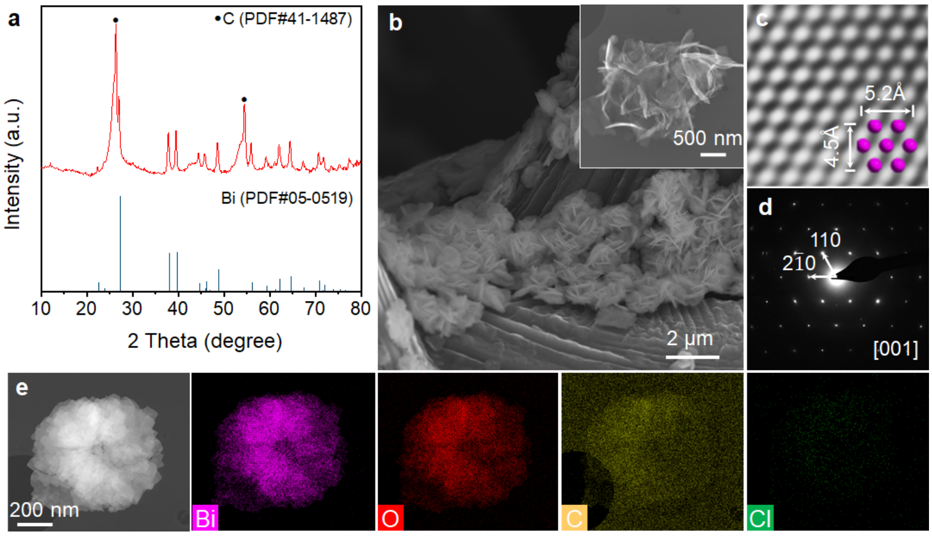

Fig. S11 (**a**) XRD pattern, (**b**) FE-SEM images, (**c**) IFFT-HRTEM image, (**d**) corresponding SAED pattern and (**e**) HAADF-STEM image and corresponding EDX element mapping images of the BiOCl-NSs derived Bi at E_{App} = -1.2 V vs RHE

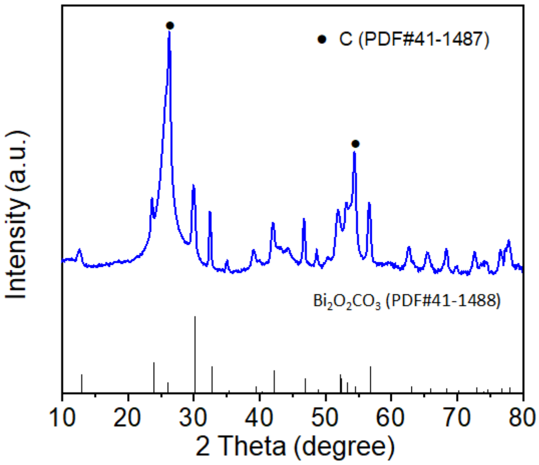

Fig. S12 XRD pattern of the as-synthesized $Bi_2O_2CO_3$

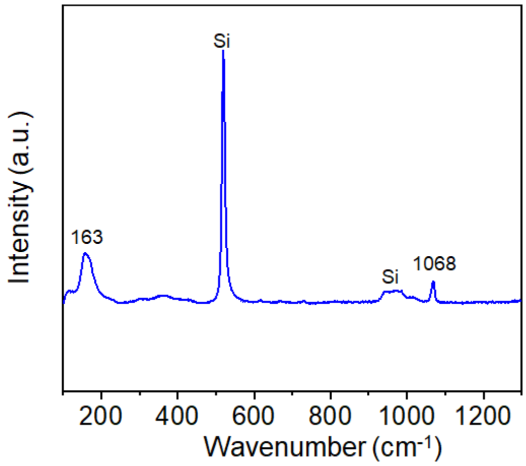

Fig. S13 Raman spectrum of the as-synthesized $Bi_2O_2CO_3$

The Raman band observed at 163 cm^{-1} can be attributed to the external vibration mode of the CO_3^{2-} group [S2], and the peak at 1068 cm^{-1} is assignable to the internal vibration v_1 of the CO_3^{2-} ion in the interlayer [S3].

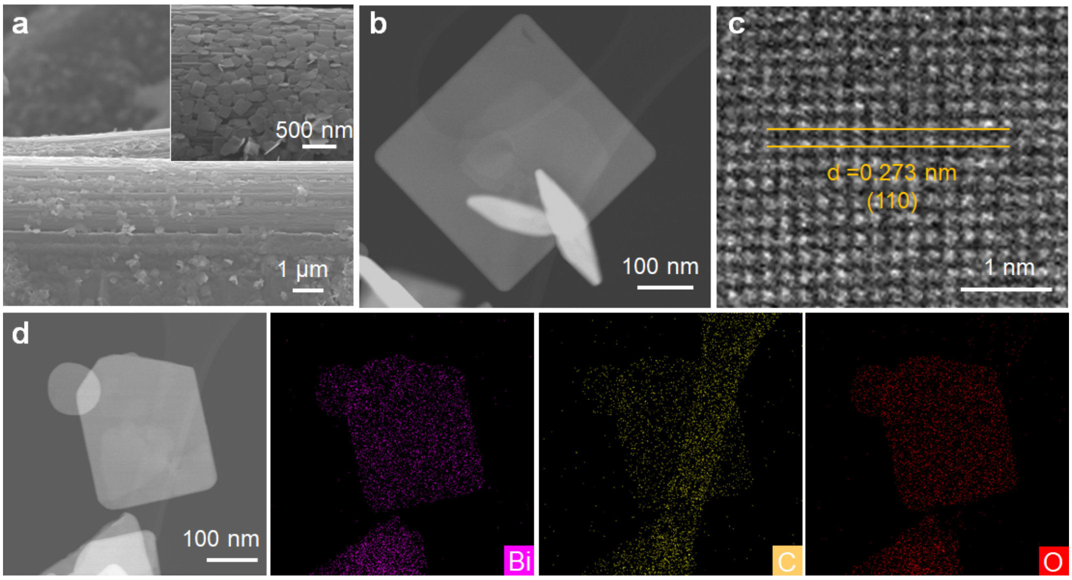

Fig. S14 (**a**) FESEM images, (**b**) TEM image, (**c**) HRTEM image and (**e**) HAADF-STEM image and corresponding EDX element mapping images of the as-synthesized $Bi_2O_2CO_3$

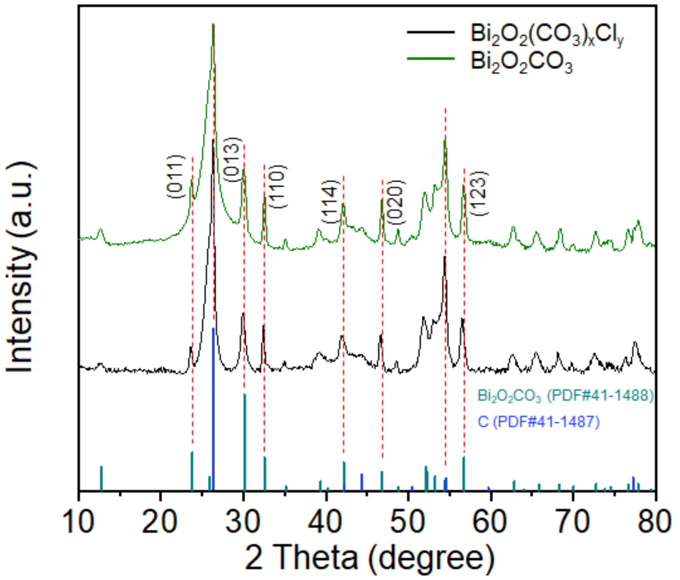

Fig. S15 XRD patterns of $Bi_2O_2(CO_3)_xCl_y$ and $Bi_2O_2CO_3$

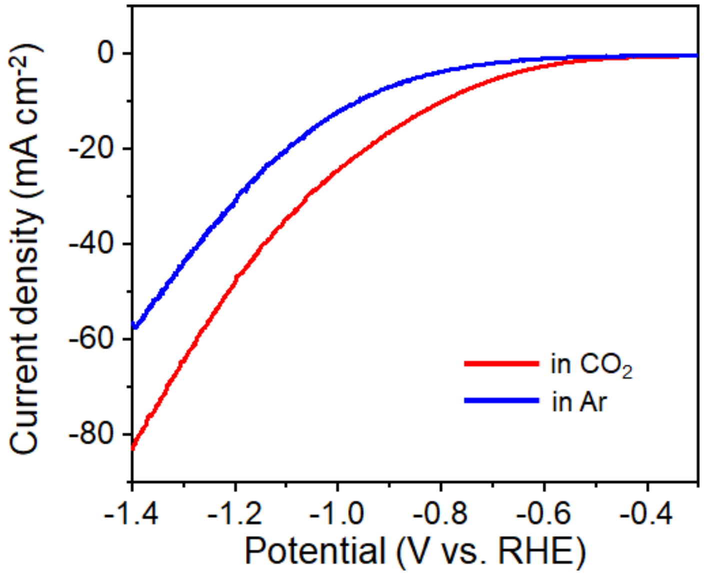

Fig. S16 LSV curves of $Bi_2O_2(CO_3)_xCl_y$ in CO_2- and Ar-saturated 0.5 M $KHCO_3$ electrolyte at a scan rate of 5 mV s^{-1}

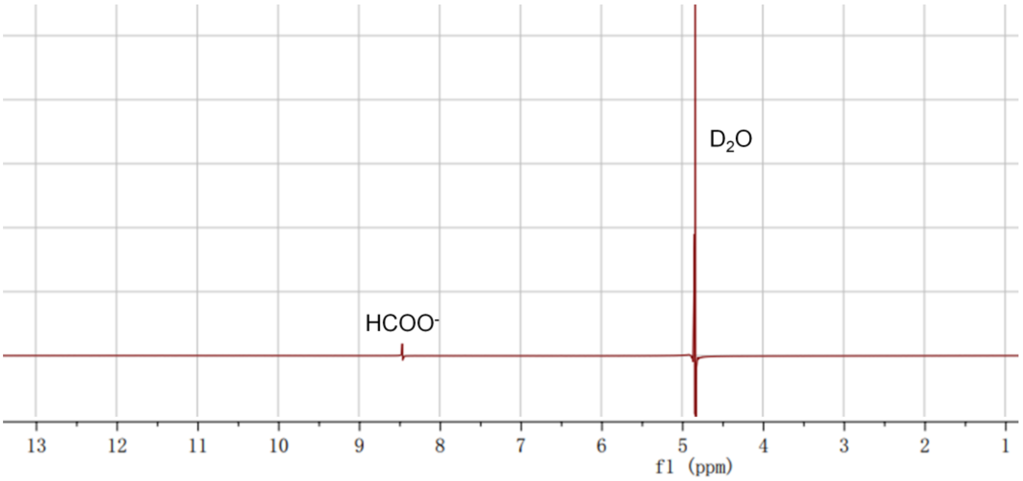

Fig. S17 NMR spectrum of $HCOO^-$ standard sample

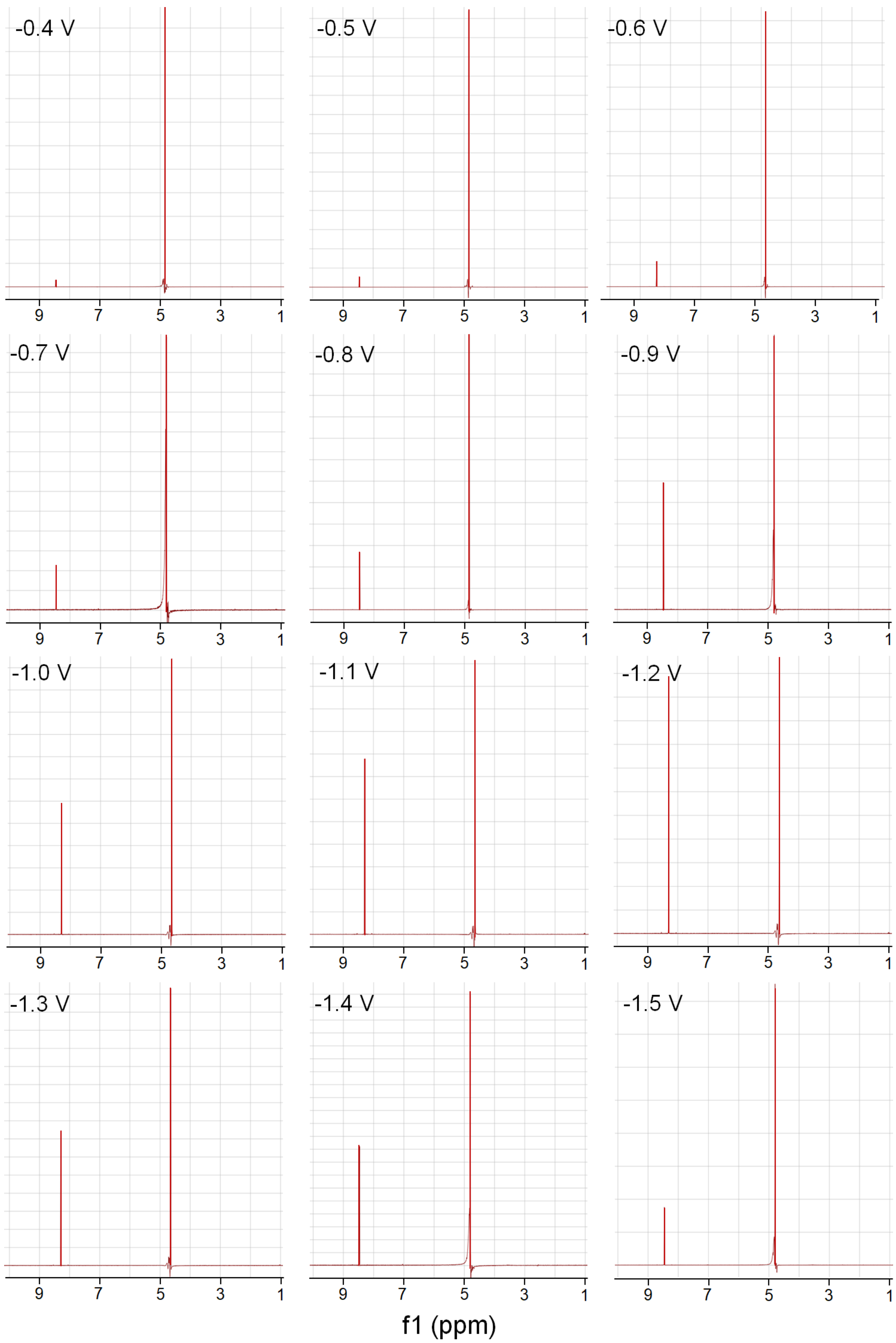

Fig. S18 NMR spectra of 0.5 M $KHCO_3$ electrolyte after CO_2 reduction at different potentials

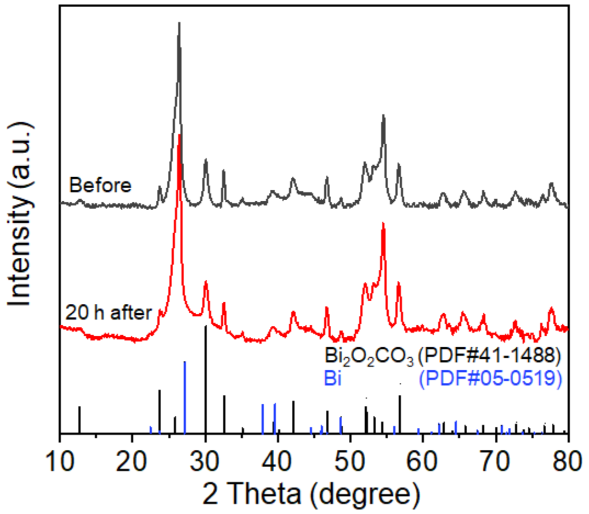

Fig. S19 XRD patterns of $Bi_2O_2(CO_3)_xCl_y$ before and after stability test at -0.8 V (vs RHE) for 20 h

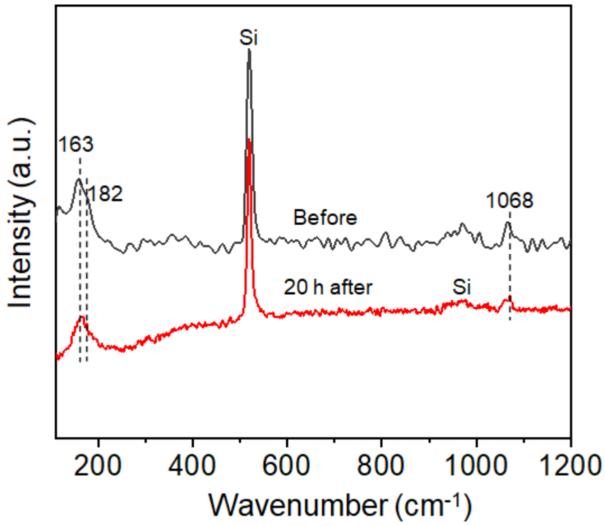

Fig. S20 Raman spectra of $Bi_2O_2(CO_3)_xCl_y$ before and after stability test at -0.8 V (vs RHE) for 20 h

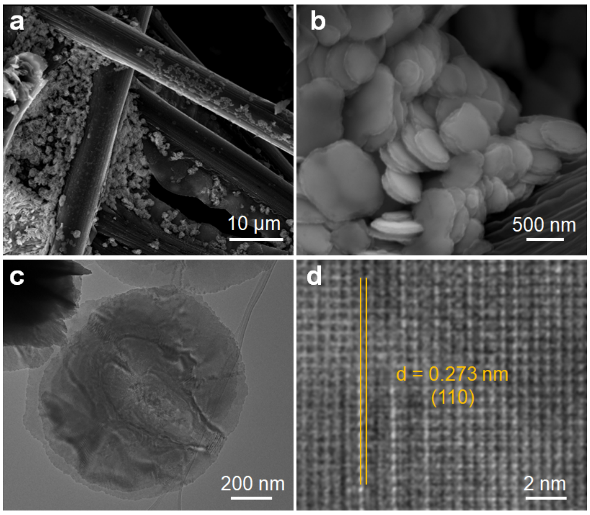

Fig. S21 (**a**, **b**) FESEM images, (**c**) TEM image, (**d**) HRTEM image of $Bi_2O_2(CO_3)_xCl_y$ after the stability test at -0.8 V (vs RHE) for 20 h

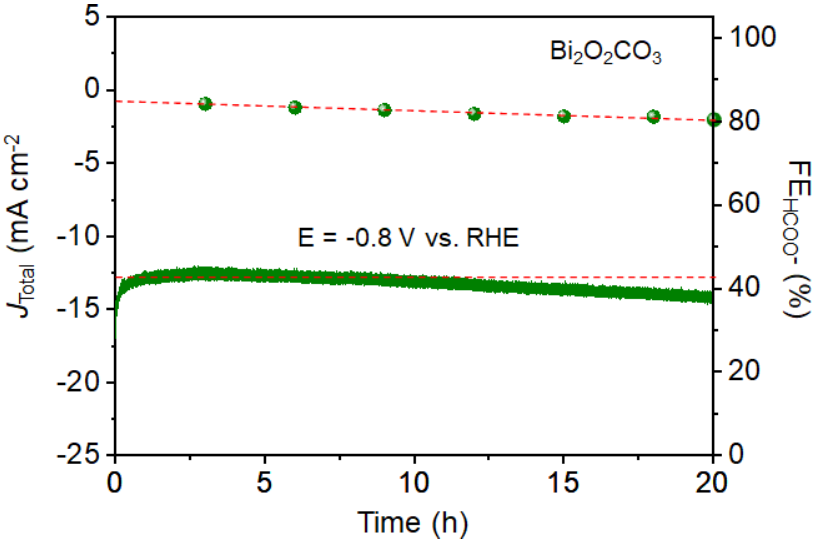

Fig. S22 Chronoamperometric curve and FE_{HCOO^-} of $Bi_2O_2CO_3$ at -0.8 V vs RHE

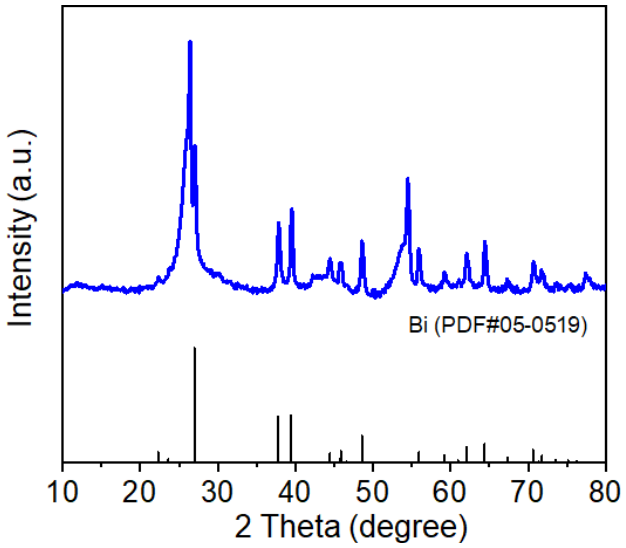

Fig. S23 XRD pattern of $Bi_2O_2CO_3$ after stability test at -0.8 V (vs RHE) for 20 h

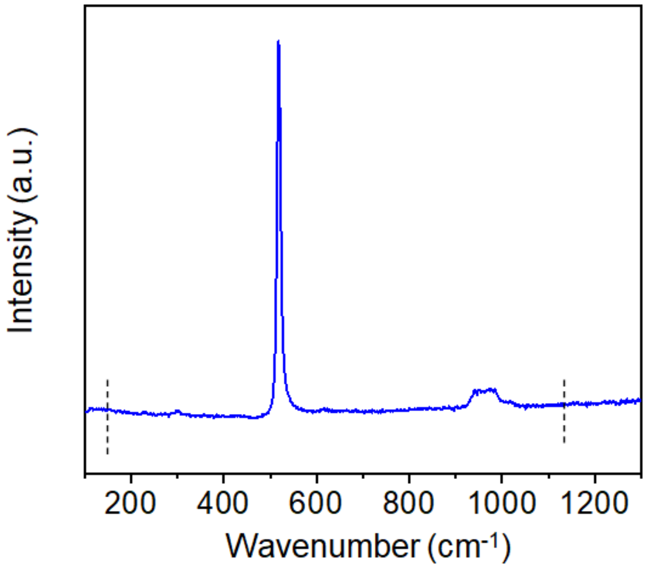

Fig. S24 Raman spectra of $Bi_2O_2CO_3$ after stability test at -0.8 V (vs RHE) for 20 h

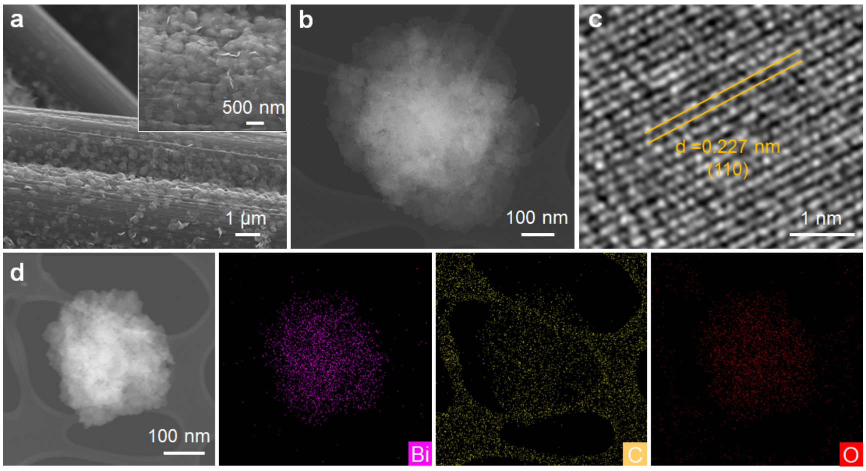

Fig. S25 (**a**) FESEM images, (**b**) TEM image, (**c**) HRTEM image and (**e**) HAADF-STEM image and corresponding element mapping images of $Bi_2O_2CO_3$ after stability test at -0.8 V (vs RHE) for 20 h

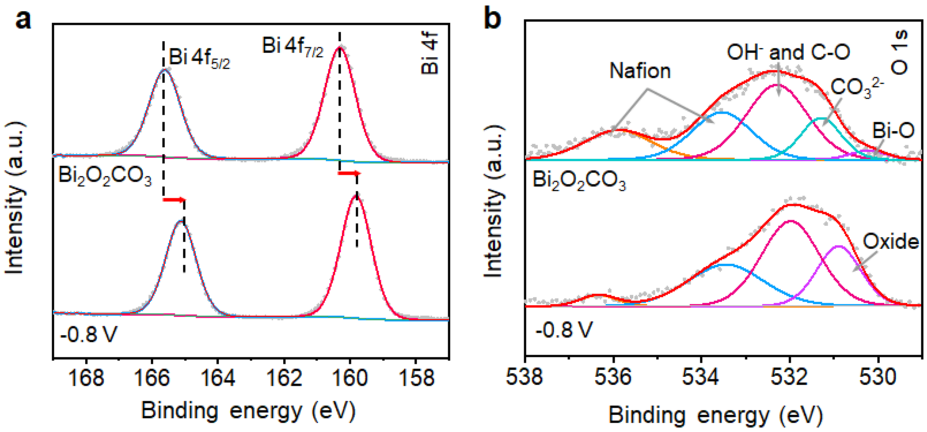

Fig. S26 High-resolution XPS spectra of (**a**) Bi 4f and (**b**) O 1s of $Bi_2O_2CO_3$ after stability test at -0.8 V vs RHE

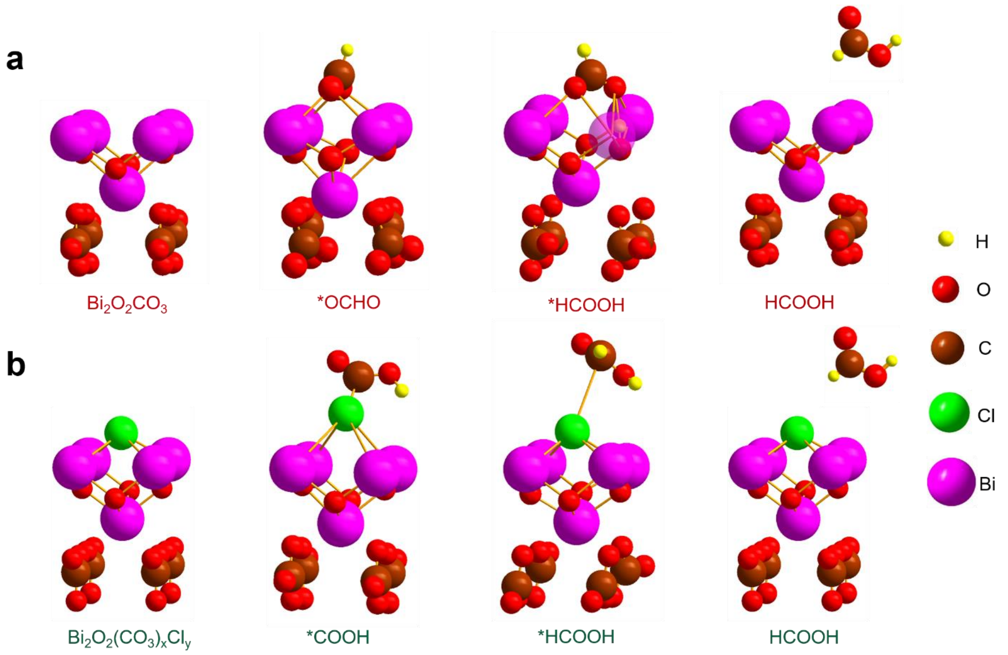

Fig. S27 DFT optimised intermediate adsorption on (**a**) $Bi_2O_2CO_3$ and (**b**) $Bi_2O_2(CO_3)_xCl_y$

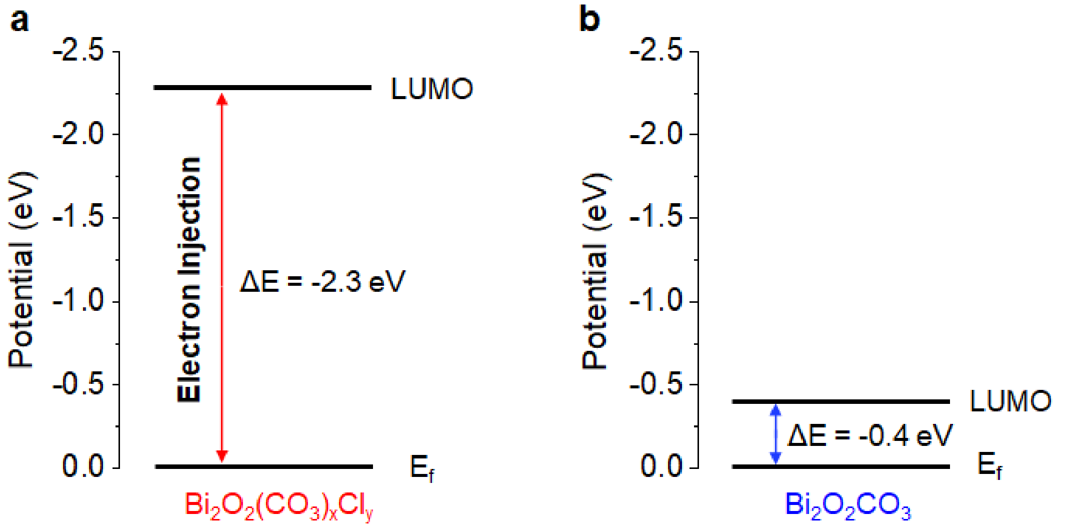

Fig. S28 Schematic illustrating the relationship between LUMO potential and the minimum potential required to reduce Bi^{3+} in (**a**) $Bi_2O_2(CO_3)_xCl_y$ and (**b**) $Bi_2O_2CO_3$ to metallic Bi°

Table S1 Reported performance of Bi-based electrocatalysts for reduction of CO_2 to $HCOO^-$

	Electrocatalysts	Electrolyte	Potential (V vs RHE)	j_{HCOO^-} (mA cm^{-2})	FE	Refs.
Metallic Bi°	Bismuthene	0.5 M $KHCO_3$	-0.58	~2.5	~98.0%	[S4]
	2D-Bi	0.5 M $KHCO_3$	-0.79	17.3	99.0%	[S5]
	Bi nanosheet	0.1 M $KHCO_3$	-1.10	16.5	86.0%	[S6]
	Bi nanoflake	0.1 M $KHCO_3$	-0.60	~2.0	100.0%	[S7]
	Bi dendrite	0.5 M $NaHCO_3$	-1.14	15.2	96.4%	[S8]
	Bi nanotube	0.5 M $KHCO_3$	-1.00	~22.0	97.0%	[S9]
	Bi dendrite	0.5 M $KHCO_3$	-0.74	2.7	89.0%	[S10]
	Dendritic Bi	0.5 M $KHCO_3$	-0.82	18.8	98.0%	[S11]
	BiNRs@NCNTs	0.1 M $KHCO_3$	-0.90	~5.0	90.9%	[S12]
	Bi-PVP/CC600	0.5 M $KHCO_3$	-0.83	~13.0	86.0%	[S13]
	SD-Bi	0.5 M $NaHCO_3$	-0.75	5.0	84.0%	[S14]
	NTD-Bi	1.0 M $KHCO_3$	-0.80	~30.0	93.0%	[S15]
	POD-Bi	0.5 M $KHCO_3$	-1.16	57.0	95.0%	[S16]
	BiOBr-templated Bi	0.1 M $KHCO_3$	-1.00	80.0	99.0%	[S17]
	BiNS	0.5 M $KHCO_3$	-1.00	22.0	100.0%	[S18]
	Bismuthene	1 M $KHCO_3$	-1.00	105.4	97.4%	[S19]
	mpBi	0.5 M $NaHCO_3$	-0.90	14.5	99.0%	[S20]
	Bi nanosheets	0.5 M $KHCO_3$	-0.80	~4.0	97.0%	[S21]
	Bi NSs	1.0 M $KHCO_3$	-0.98	75.0	95.5%	[S22]
	Bi NS	0.1M $KHCO_3$	-1.10	10.0	92.0%	[S23]
	Bi-ene	0.5 M $KHCO_3$	-0.90	~25.0	~98.6%	[S24]
Oxide	β-phase Bi_2O_3/Bi	0.1 M $KHCO_3$	-1.20	~22.5	87.0%	[S25]
	Bi_2O_3NSs@MCCM	0.1 M $KHCO_3$	-1.26	17.7	93.8%	[S26]
	Bi_2O_3-NGQDs	0.5 M $KHCO_3$	-0.90	18.0	95.6%	[S27]
	BiO_x/C	1.0 M $NaHCO_3$/NaCl	-1.07	15.0	93.4%	[S28]
	Bi_2O_3@C	0.5 M $KHCO_3$	-0.90	7.5	92.0%	[S29]
	Bi_2O_3	0.5 M $KHCO_3$	-0.90	8.0	91.0%	[S30]
Subcarbonate	$Bi_2O_2CO_3$	0.5 M $NaHCO_3$	-0.7	~11.0	85%	[S31]

Table S2 EXAFS data fitting results of $Bi_2O_2CO_3$ and $Bi_2O_2(CO_3)_xCl_y$

Sample	Shell	CN	R(Å)	$\sigma^2(10^{-3}Å^2)$	ΔE_0(eV)	R factor
$Bi_2O_2CO_3$	Bi-O	2.27 ± 0.20	2.24 ± 0.07	4.26	-2.649	0.0484
	Bi-C	1.75 ± 0.83	3.36 ± 0.02	4.26		
$Bi_2O_2(CO_3)_xCl_y$	Bi-O	2.34 ± 0.21	2.22 ± 0.01	4.97	-5.620	0.0321
	Bi-C	1.51 ± 1.24	3.38 ± 0.06	4.97		
	Bi-Cl	0.33 ± 0.41	3.11 ± 0.09	9.69		

Note the ~ 5 Å difference between the fitted R values and the peaks in FT-EXAFS spectrum in R space. Single scattering paths of $Bi_2O_2CO_3$ and the Bi-Cl path from the optimized Cl adsorbed $Bi_2O_2CO_3$(001) structure are used for the fitting.

Supplementary References

[S1] J. Lu, W. Zhou, X. Zhang, G. Xiang, Electronic structures and lattice dynamics of layered biocl single crystals. J. Phys. Chem. Lett. **11**(3), 1038-1044 (2020). https://doi.org/10.1021/acs.jpclett.9b03575

[S2] G.E. Tobon-Zapata, S.B. Etcheverry, E.J. Baran, Vibrational spectrum of bismuth subcarbonate. J. Mater. Sci. Lett. **16**, 656-657 (1997). https://doi.org/10.1023/A:1018527602604

[S3] O.F. Lopes, K.T.G. Carvalho, W. Avansi, D.M.B. Milori, C. Ribeiro, Insights into the photocatalytic performance of $Bi_2O_2CO_3/BiVO_4$ heterostructures prepared by one-step hydrothermal method. RSC Adv. **8**(20), 10889-10897 (2018). https://doi.org/10.1039/c8ra00605a

[S4] F. Yang, A.O. Elnabawy, R. Schimmenti, P. Song, J. Wang et al., Bismuthene for highly efficient carbon dioxide electroreduction reaction. Nat. Commun. **11**, 1088 (2020). https://doi.org/10.1038/s41467-020-14914-9

[S5] C. Xia, P. Zhu, Q. Jiang, Y. Pan, W. Liang et al., Continuous production of pure liquid fuel solutions via electrocatalytic CO_2 reduction using solid-electrolyte devices. Nat. Energy **4**, 776-785 (2019). https://doi.org/10.1038/s41560-019-0451-x

[S6] W. Zhang, Y. Hu, L. Ma, G. Zhu, P. Zhao et al., Liquid-phase exfoliated ultrathin Bi nanosheets: uncovering the origins of enhanced electrocatalytic CO_2 reduction on two-dimensional metal nanostructure. Nano Energy **53**, 808-816 (2018). https://doi.org/10.1016/j.nanoen.2018.09.053

[S7] S. Kim, W.J. Dong, S. Gim, W. Sohn, J.Y. Park et al., Shape-controlled bismuth nanoflakes as highly selective catalysts for electrochemical carbon dioxide reduction to formate. Nano Energy **39**, 44-52 (2017). https://doi.org/10.1016/j.nanoen.2017.05.065

[S8] H. Zhong, Y. Qiu, T. Zhang, X. Li, H. Zhang et al., Bismuth nanodendrites as a high performance electrocatalyst for selective conversion of CO_2 to formate. J. Mater. Chem. A **4**(36), 13746-13753 (2016). https://doi.org/10.1039/c6ta06202d

[S9] K. Fan, Y. Jia, Y. Ji, P. Kuang, B. Zhu et al., Curved surface boosts electrochemical CO_2 reduction to formate via bismuth nanotubes in a wide potential window. ACS Catal. **10**(1), 358-364 (2019). https://doi.org/10.1021/acscatal.9b04516

[S10] J.H. Koh, D.H. Won, T. Eom, N.K. Kim, K.D. Jung et al., Facile CO_2 electro-reduction to formate via oxygen bidentate intermediate stabilized by high-index planes of Bi dendrite catalyst. ACS Catal. **7**(8), 5071-5077 (2017). https://doi.org/10.1021/acscatal.7b00707

[S11] M. Fan, S. Prabhudev, S. Garbarino, J. Qiao, G.A. Botton et al., Uncovering the nature of electroactive sites in nano architectured dendritic Bi for highly efficient CO_2 electroreduction to formate. Appl. Catal. B Environ. **274**, 119031 (2020). https://doi.org/10.1016/j.apcatb.2020.119031

[S12] W. Zhang, S. Yang, M. Jiang, Y. Hu, C. Hu et al., Nanocapillarity and nanoconfinement effects of pipet-like bismuth@carbon nanotubes for highly efficient electrocatalytic CO_2 reduction. Nano Lett. **21**(6), 2650-2657 (2021). https://doi.org/10.1021/acs.nanolett.1c00390

[S13] D. Wu, X. Wang, X.Z. Fu, J.L. Luo, Ultrasmall Bi nanoparticles confined in carbon nanosheets as highly active and durable catalysts for CO_2 electroreduction. Appl. Catal. B Environ. **284**, 119723 (2021). https://doi.org/10.1016/j.apcatb.2020.119723

[S14] Y. Zhang, F. Li, X. Zhang, T. Williams, C.D. Easton et al., Electrochemical reduction of CO_2 on defect-rich Bi derived from Bi_2S_3 with enhanced formate selectivity. J. Mater. Chem. A **6**(11), 4714-4720 (2018). https://doi.org/10.1039/c8ta00023a

[S15] Q. Gong, P. Ding, M. Xu, X. Zhu, M. Wang et al., Structural defects on converted bismuth oxide nanotubes enable highly active electrocatalysis of carbon dioxide reduction. Nat. Commun. **10**, 2807 (2019). https://doi.org/10.1038/s41467-019-10819-4

[S16] S. He, F. Ni, Y. Ji, L. Wang, Y. Wen et al., The p-orbital delocalization of main-group metal boosting CO_2 electroreduction. Angew. Chem. Int. Ed. **57**(49), 16114-16119 (2018). https://doi.org/10.1002/anie.201810538

[S17] F.P.G. Arquer, O.S. Bushuyev, P.D. Luna, C.T. Dinh, A. Seifitokaldani et al., 2D metal oxyhalide-derived catalysts for efficient CO_2 electroreduction. Adv. Mater. **30**(38), 1802858 (2018). https://doi.org/10.1002/adma.201802858

[S18] N. Han, Y. Wang, H. Yang, J. Deng, J. Wu et al., Ultrathin bismuth nanosheets from in situ topotactic transformation for selective electrocatalytic CO_2 reduction to formate. Nat. Commun. **9**, 1320 (2018). https://doi.org/10.1038/s41467-018-03712-z

[S19] W. Ma, J. Bu, Z. Liu, C. Yan, Y. Yao et al., Monoclinic scheelite bismuth vanadate derived bismuthene nanosheets with rapid kinetics for electrochemically reducing carbon dioxide to formate. Adv. Funct. Mater. **31**(4), 2006704 (2020). https://doi.org/10.1002/adfm.202006704

[S20] H. Yang, N. Han, J. Deng, J. Wu, Y. Wang et al., Selective CO_2 reduction on 2D mesoporous Bi nanosheets. Adv. Energy Mater. **8**(35), 1801536 (2018). https://doi.org/10.1002/aenm.201801536

[S21] M. Zhao, Y. Gu, W. Gao, P. Cui, H. Tang et al., Atom vacancies induced electron-rich surface of ultrathin Bi nanosheet for efficient electrochemical CO_2 reduction. Appl. Catal. B Environ. **266**, 118625 (2020). https://doi.org/10.1016/j.apcatb.2020.118625

[S22] J. Yang, X. Wang, Y. Qu, X. Wang, H. Huo et al., Bi-based metal-organic framework derived leafy bismuth nanosheets for carbon dioxide electroreduction. Adv. Energy Mater. **10**(36), 2001709 (2020). https://doi.org/10.1002/aenm.202001709

[S23] D. Yao, C. Tang, A. Vasileff, X. Zhi, Y. Jiao et al., The controllable reconstruction of Bi-MOFs for electrochemical CO_2 reduction through electrolyte and potential mediation. Angew. Chem. Int. Ed. **60**(33), 18178-18184 (2021). https://doi.org/10.1002/anie.202104747

[S24] C. Cao, D.D. Ma, J.F. Gu, X. Xie, G. Zeng et al., Metal-organic layers leading to atomically thin bismuthene for efficient carbon dioxide electroreduction to liquid fuel.

Angew. Chem. Int. Ed. **132**(35), 15124-15130 (2020). https://doi.org/10.1002/anie.202005577

[S25] T. Tran-Phu, R. Daiyan, Z. Fusco, Z. Ma, R. Amal et al., Nanostructured β-Bi_2O_3 fractals on carbon fibers for highly selective CO_2 electroreduction to formate. Adv. Funct. Mater. **30**(3), 1906478 (2019). https://doi.org/10.1002/adfm.201906478

[S26] S. Liu, X.F. Lu, J. Xiao, X. Wang, X.W.D. Lou, Bi_2O_3 nanosheets grown on multi-channel carbon matrix catalyze efficient CO_2 electroreduction to HCOOH. Angew. Chem. Int. Ed. **58**(39), 13828-13833 (2019). https://doi.org/10.1002/anie.201907674

[S27] Z. Chen, K. Mou, X. Wang, L. Liu, Nitrogen-doped graphene quantum dots enhance the activity of Bi_2O_3 nanosheets for electrochemical reduction of CO_2 in a wide negative potential region. Angew. Chem. Int. Ed. **57**(39), 12790-12794 (2018). https://doi.org/10.1002/anie.201807643

[S28] C.W. Lee, J.S. Hong, K.D. Yang, K. Jin, J.H. Lee et al., Selective electrochemical production of formate from carbon dioxide with bismuth-based catalysts in an aqueous electrolyte. ACS Catal. **8**(2), 931-937 (2018). https://doi.org/10.1021/acscatal.7b03242

[S29] P. Deng, F. Yang, Z. Wang, S. Chen, Y. Zhou et al., Metal-organic frameworks-derived carbon nanorods encapsulated bismuth oxides for rapid and selective CO_2 electroreduction to formate. Angew. Chem. Int. Ed. **59**(27), 10807-10813 (2020). https://doi.org/10.1002/anie.202000657

[S30] P. Deng, H. Wang, R. Qi, J. Zhu, S. Chen et al., Bismuth oxides with enhanced bismuth-oxygen structure for efficient electrochemical reduction of carbon dioxide to formate. ACS Catal. **10**(1), 743-750 (2019). https://doi.org/10.1021/acscatal.9b04043

[S31] Y. Zhang, X. Zhang, Y. Ling, F. Li, A.M. Bond et al., Controllable synthesis of few-layer bismuth subcarbonate by electrochemical exfoliation for enhanced CO_2 reduction performance. Angew. Chem. Int. Ed. **57**(40), 13283-13287 (2018). https://doi.org/10.1002/ange.201807466
